# COVID-19 spread algorithm in the international airport network-DetArpds

**DOI:** 10.7717/peerj-cs.1228

**Published:** 2023-02-16

**Authors:** Cesar Guevara, Dennys Coronel, Byron Eduardo Salazar Maldonado, Jorge Eduardo Salazar Flores

**Affiliations:** 1 DataLab, The Institute of Mathematical Sciences (ICMAT-CSIC), Madrid, Spain; 2 Centre of Mechatronics and Interactive Systems (MIST), Universidad Tecnológica Indoamérica, Quito, Pichincha, Ecuador; 3 Neurosurgery Department, Hospital de las Fuerzas Armadas HE-1, Quito, Pichincha, Ecuador; 4 Neurosurgery Department, Metropolitano Hospital, Quito, Pichincha, Ecuador

**Keywords:** COVID-19, Algorithm, Spread, Airport, Network, Routes

## Abstract

Due to COVID-19, the spread of diseases through air transport has become an important issue for public health in countries globally. Moreover, mass transportation (such as air travel) was a fundamental reason why infections spread to all countries within weeks. In the last 2 years in this research area, many studies have applied machine learning methods to predict the spread of COVID-19 in different environments with optimal results. These studies have implemented algorithms, methods, techniques, and other statistical models to analyze the information in accuracy form. Accordingly, this study focuses on analyzing the spread of COVID-19 in the international airport network. Initially, we conducted a review of the technical literature on algorithms, techniques, and theorems for generating routes between two points, comprising an analysis of 80 scientific papers that were published in indexed journals between 2017 and 2021. Subsequently, we analyzed the international airport database and information on the spread of COVID-19 from 2020 to 2022 to develop an algorithm for determining airport routes and the prevention of disease spread (DetARPDS). The main objective of this computational algorithm is to generate the routes taken by people infected with COVID-19 who transited the international airport network. The DetARPDS algorithm uses graph theory to map the international airport network using geographic allocations to position each terminal (vertex), while the distance between terminals was calculated with the Euclidian distance. Additionally, the proposed algorithm employs the Dijkstra algorithm to generate route simulations from a starting point to a destination air terminal. The generated routes are then compared with chronological contagion information to determine whether they meet the temporality in the spread of the virus. Finally, the obtained results are presented achieving a high probability of 93.46% accuracy for determining the entire route of how the disease spreads. Above all, the results of the algorithm proposed improved different computational aspects, such as time processing and detection of airports with a high rate of infection concentration, in comparison with other similar studies shown in the literature review.

## Introduction

Air transport systems have become a promising area of research due to the latest developments in the spread of global pandemics. Air terminals are a meeting point for hundreds of thousands of people from different parts of the world, which facilitates the direct or indirect transmission of various diseases (Derjany et al., 2020; Huber & Rinner, 2020; Nikolaou & Dimitriou, 2020). Over the last 20 years, many different viruses have spread through different countries *via* airport networks. One of the well-known cases was the 2014 Ebola outbreak, which started in West Africa and caused more than 11,000 deaths worldwide (Bailey et al., 2018; Tanade et al., 2019). Another notable case was the H1N1 virus, which was initiated in 2009 in Mexico and proceeded to spread in more than 70 countries, causing nearly 650,000 deaths between 2009 and 2010 (Marziano et al., 2017; Ng et al., 2019). Another important case was the MERS-CoV virus, which was first detected in Saudi Arabia in 2012. This virus caused more than 2,566 infections and 882 deaths. Finally, the SARS virus started in China in November 2002 and spread to more than 26 countries, causing 8,098 confirmed infections and 774 deaths (Jothimani et al., 2020).

The main objectives of this research are as follows:

- Analyze the spread of COVID-19 through international air terminals from the origin airport to the destination.
- Determine the possible routes by which the virus could spread and identify terminals with high concentrations of air traffic that might have led to contagion.
- Analyze the application of graph theory to generate an interconnected network of international airports and identify multiple routes of contagion using Dijkstra’s algorithm.
- Verify the simulated routes and compare them with the chronological data of COVID-19 infections to determine the routes with the highest probability of spreading the virus.
- Determine and identify as an optimal solution for the application of both techniques in a simulated environment to evaluate the results obtained with accurate pandemia data (COVID-19 spread data).

This type of study is essential for helping the decision-making of governments and public health organizations to reduce the transmission of highly contagious diseases in the population. In addition, this article proposes an essential tool for preventing future pandemics in an automatized way. Finally, this study opens a crucial issue: the application of data science, biology, medicine, and government management to prevent the spread of infectious diseases.

The rest of this document is organized as follows “Related work” describes related work, which serves as a fundamental basis for developing the proposal. In “Materials”, the study materials are presented, wherein the databases used for developing the proposal are detailed. “COVID-19 spread analysis” presents the proposed algorithm, with its structure and operation, while the results obtained and comparisons with other relevant works are presented in “Results and discussion”. Finally, the conclusions and future work identified in the research are presented in “Conclusions and future work”.

## Related work

We conducted, a bibliographic study on the most commonly used algorithms for the generation of routes in air terminals, with a special emphasis on the spread of diseases. The search was restricted to articles published in the last 5 years (2017–2021), generating 80 articles from the following publishers: Springer (18 articles), Wiley Online Library (one), Elsevier (24 articles), IEEE (26 articles), Nature (one article), Acm (four articles), and Mdpi (six articles). The articles were then classified according to the type of contribution: conferences (60 articles) and journals (20 articles).

### Evolutionary algorithms

The application of evolutionary algorithms (EAs) has been widespread in the specialized literature on the generation of routes. The study by Ho-Huu et al. (2018) proposed a method to solve route optimization problems with considerable noise using an EA called MOEA/D. The aim of this research was to generate new optimal air routes for noise reduction at Rotterdam Airport. The proposed method achieved high robustness and reliability. In addition, it generated different route replacement strategies, stop condition criteria, and constraint management to enable better decision-making.

Kammoun & Rezg (2018) developed a hybrid system for routing and reprogramming aircraft using a genetic algorithm and timed Petri net. The goal of this study was to minimize the total routing time and the amount of flight rescheduling. The results were encouraging because the system facilitated the management of many flights (including rescheduling) to improve their handling, rendering the processing time more efficient.

Wu et al. (2018) posited a genetic classification algorithm called DA-NSGA-II, to solve the problem of airspace congestion (traffic flow) in China. The objective of this study was to analyze the degree of air traffic congestion, total cost of operations, flight delay times, and all military and civilian flights. The results were optimal, with the DA-NSGA-II model generating improved air routes in a short execution time with a low degree of crossing between aircraft. Alieksieiev (2019), analyzed the problem of routing in airspace by using an EA combined with free routing airspace (FRA) and route availability document methods for better air routing.

Turky et al. (2020) presented a memetic EA that identified the shortest route based on the expected travel time. This method produces a prediction that determines travel times and then optimizes the shortest path by applying the memetic algorithm and deep learning. This method considers factors such as weather conditions, traffic, time of day, and day of the week. The results were satisfactory because the proposed method was highly effective compared to the most advanced procedures.

Cai et al. (2020), analyzed the improvement in air traffic network (ATN) robustness. The study proposed eliminating ATN edges using the Braess paradox, which employs an unmastered genetic classification algorithm (NSGA-II). The results were optimal because they improved robustness by 100% by eliminating fewer than six edges, while the remaining six edges yielded approximately 10% improvement.

Yanzhe & Bingxiang (2020) presented a model for evaluating and predicting the spread of COVID-19 in four countries (China, Italy, Great Britain, and the United States) using the ant colony algorithm and SIR (susceptible, infectious, or recovered) model. The objectives of their study were to analyze the epidemiological situation of COVID-19 and to examine the trend of the spread of the virus. The results of their proposed model were efficient because it reliably predicted the epidemiological trend, helping government decision-making and reducing infection rates and the spread of the pandemic. In addition, the research confirmed that reducing mass travel and closing severely affected areas could effectively slow the speed of the COVID-19 outbreak.

Weerasinghe et al. (2020), proposed a system for optimizing the daily operations of airlines through genetic programming. The objective of this research was to determine optimal flight routes, fleet allocation, gate assignment, and crew assignment. The results were satisfactory because the proposed system obtained reasonable results in a short period. In addition, the system contributed to the optimization of decision-making in Airline Operations Control Centers (AOCCs).

A study carried out by Fijar Awalivian, Suyanto & Sa’Adah (2021) detailed the serious problem of the spread of the COVID-19 virus, which affected flight scheduling and passenger-carrying capacity. In this study, the multi-objective antlion optimization (MALO) method was implemented, which solved flight scheduling and aircraft redirection problems under the current pandemic conditions. The results obtained were optimal because the MALO method can support large programming tasks by quickly converging a large dataset.

The algorithm used real data from the International Civil Aviation Organization (2021) for testing. The results were encouraging, as they demonstrated a good focus on finding a route between the departure and destination points and implemented a better strategy to generate improved short or optimal routes.

Cai, Ang & Alam (2021) presented a method to reduce the risk of collisions on Singapore’s air routes. This method used two EAs: NSGA-II and NSGA-III. The objective of the study was to analyze the risk of collision in each traffic flow. The results were optimal, and the research contributed to air traffic management both strategically and tactically.

### Dynamic programming

Azis et al. (2018) presented clear examples of a dynamic programming (DP) algorithm, in which they compared conventional and heuristic methods to solve the problem of the shortest path. The data used for these studies were obtained from the Losari Beach route. The results were optimal since the conventional method provided a not better solution (shorter time) compared to the heuristic method. Moreover, the conventional method provided a less optimal local route.

Prakash, Piplani & Desai (2018) proposed an algorithm to manage aircraft on a single runway through DP. The aim of the study was to maximize aircraft entry and exit performance. The results demonstrated that the algorithm was easy to implement, maximized the operational performance of aircraft control, and provided optimal solutions in real time.

Brown, Hirabayshi & Wickramasinghe (2019) presented a model for air route planning that considers wind factors for medium-and long-haul flights. The objective of this study was to generate, draw, and optimize air trajectories through dynamic programming on the free route of the North Pacific. The results were effective because the proposed method provided several alternate routes for the aircraft. Moreover, this method facilitated the calculation of optimal trajectories (shorter routes) with shorter flight times and minimum fuel consumption (based on the wind variable).

Tian et al. (2019) provided details of a model for minimizing the ecological operating costs (GDOC) of cruise aircraft. The objective of this study was to analyze fuel consumption, flight time, and distance at the cruise stage using DP. The results were encouraging since the method generated a sustainable optimal flight plan for the environment by reducing altitude and speed. In addition, the model minimized the operating costs of civil aviation. Yu & Wang (2019) produced another interesting article in the same area of DP. They proposed a model to improve the efficiency of allocating connecting flights in China through dynamic scheduling. The main objective of the study was to collect data such as flight times and optimal flight routes. The results indicated that the proposed model maximized the economic benefits of the airlines by optimizing flight times.

Chen & Solak (2020) conducted another recent study in the same context for developing a flight management model for the Metropolitan Airport of Wayne County in Detroit (United States). This model employed DP to determine the optimal number of aircraft that could be directed to the runway tail and to identify optimal output-measurement policies. The results indicated that the model improved the efficiency of air traffic management and outbound operations, helping to minimize the overall costs for airlines.

Ntakolia, Caceres & Coletsos (2020), presented a model for air traffic management that used DP. The aim of this study was to improve aircraft traffic safety and increase the efficiency of the air network. The results were acceptable, as they generated optimal flight paths based on the variables of flight delays, flight durations, ground waiting delays, flight cancellations, flight speed deviations, and flight level alterations.

Recently, Yi, Tong & LiuXi (2020) detailed a spatial digital grid model for monitoring short-term flight conflicts in real time. The objective of this study was to compare the grid coordinates of each node, determine the grid coordinates of the nodes in possible collisions, and classify the type of collision using DP. The results were optimal because a reliable prediction of possible collisions was generated. In addition, the model suggested possible optimal solutions for conflict resolution through the strategic selection of maneuvers according to the performance index.

Ahmed, Bousson & Coelho (2021) proposed a modified dynamic programming (MDP) approach to flight path optimization. The objective of this research was to generate optimal air routes with minimum fuel consumption, and the results obtained were successful. By using MDP, they generated optimal routes that improved flight efficiency by reducing fuel consumption by 10.1% and yielded a 10.99% reduction in aircraft gas emissions.

In the latest study conducted in this field by de Campos, Vieira & Munari (2021), a method for the routing of aircraft with crew assignment was presented using DP. The objective of this study was to generate routes with lower operating costs. The results were acceptable, with the model generating optimal routes that reduced operating costs by 23%.

### Dijkstra algorithm

Dijkstra’s algorithm (DJK) uses graphs to determine the shortest path based on the weight of each edge. An example application of this algorithm was presented by Adacher & Flamini (2018). In this study, the direct costs of robust routes were analyzed with respect to the shorter routes to reduce congestion and air delays by applying Dijkstra’s algorithm. Moreover, a robust route algorithm was developed to calculate robust air routes based on a real traffic network database obtained from Flightradar (2021) study. The study obtained a valid alternative route for each network point, which allowed aircraft to reach their destination within a certain time limit, with optimal results. One of the most relevant works in the application of Dijkstra’s algorithm was by Gao et al. (2018), in which a method was proposed for solving the problem of the shortest path in a network of domestic airlines in China. This study was based on Dijkstra’s algorithm, Fenwick’s tree algorithm, and the analytic hierarchy process (AHP), with the aim of quantitatively evaluating the accessibility of airports. The proposed method used data provided by the Chinese airline network from July 1, 2016, to July 7, 2016. The study concluded that the proposed method could become a guide for airlines and civil aviation administration for further development and management.

Xiao, Cai & Abbass (2018) presented a hybrid algorithm of indirect and direct coding to solve the problem of air traffic flow in the Chinese network. This algorithm controlled the weights in each arc to select optimal flight paths based on the current air traffic. It was based on a heuristic technique using Dijkstra’s algorithm to generate different types of routes. The results obtained from the proposed method demonstrated that it surpassed the direct coding method in terms of efficiency and effectiveness.

An interesting article in the same area was presented by Dabachine, Bouikhalene & Balouki (2019), in which a model for aircraft routing at Swiftair Casablanca Airport was proposed using Dijkstra’s algorithm. The aim of this study was to reduce flight delays and overall travel costs. The model provided different optimal paths in a short computational time, facilitated the planning of trajectories, and supported real-time decision-making.

Petrov (2019) presented a method for routing aircraft at low altitudes using Dijkstra’s algorithm, the distance, and the magnitude of height change. The results were acceptable, and the proposed method generated hidden routing through a mountain range and a route that avoided radar detection zones.

Dudi et al. (2020) presented a robust methodology called Airspace Map, which was aimed at planning routes with high aircraft safety (crew members and passengers) in unfamiliar environments. Their methodology was based on Dijkstra’s algorithm and Voronoi’s diagram. The results demonstrated that the Airspace Map helps avoid redundant obstacles in the planning of air trajectories in a short time and with adequate precision. Similarly, Jazzar et al. (2020) developed a methodology for generating shorter routes with low cost using Dijkstra’s algorithm. This methodology used real data from the route between Beirut and New York City. The results were encouraging, as the system generated different optimal routes. This methodology can help decision-making, cost saving, and travel time reduction.

Zhao et al. (2020) proposed a method for preventing air traffic collisions using Dijkstra’s algorithm. The objective of this study was to create a collision risk map. The results indicated that the model was efficient and safe, generating routes with minimum cost and a low probability of collision.

Finally, Maristany de las Casas, Sedeño-Noda & Borndörfer (2021), developed a new algorithm called the multi-objective Dijkstra algorithm (MDA), which solved the problem of the multi-objective shortest path. Through the simplification of tags, it parallelized some subpaths, facilitating the calculation of a complete minimum set of efficient paths for a given instance. The data used for this research were provided by Airway Networks. The results of the proposed MDA algorithm provided a response time that was improved by a factor of between 2 and 9 compared to Martins’ algorithm. Weiszer, Burke & Chen (2020), proposed an aerial routing algorithm called NAMOA, which determined the shortest route in ground operations based on multi-target trajectories. The study applied heuristic functions based on cost preferences, running time, and fuel consumption. The algorithm was developed in Python with a set of real data from Doha International (DOH), Hong Kong International (HKG), and Beijing Capital International (PEK) airports. The results were encouraging, with the algorithm finding a set of optimal solutions in a single run in a short time.

### Neural networks

Lin, Wei Zhang & Liu (2019), presented a model called ConvLSTM, which analyzed the spatial traffic characteristics and predicted air traffic flow with real data. This model was based on an end-to-end deep-learning approach using a CNN and a recurrent neural network. The results indicated that the model predicted the distribution of traffic flow at different flight levels accurately and stably.

In another work on neural networks by Liu et al. (2019), a model was proposed based on a deep neural network (R-3DCNN) for air traffic management. The results were optimal, and the proposed model incorporated temporal and spatial dependencies of air traffic flow, providing solid and accurate predictions pertaining to the distribution of flight levels.

Wang et al. (2019) proposed a system for the detection and resolution of conflicts in air traffic in China based on reinforcement deep learning (DRL). The results were acceptable since the system generated an optimized trajectory within 200 ms while avoiding conflicts and changes in the angles of the course of the aircraft.

Han et al. (2019) developed a method for predicting aerial trajectories using a gated recurrent unit (GRU) neural network. The objective of this study was to accurately generate air routes that guarantee safety and efficiency in air traffic operations. The results indicated that the method could generate reliable real-time predictions with high accuracy.

Wang et al. (2020), analyzed the spread and identification of highly suspected cases of COVID-19 based on the Internet of Things (loT) and graph theory with a reinforced learning approach. The results were encouraging and could help decision-making to effectively reduce the rate of epidemiological reproduction of the infection. In addition, the use of these techniques could help in the early identification of COVID-19 cases. On similar lines, Ma & Tian (2020) developed a trajectory prediction method based on the aerodynamics of a ship. This method used deep learning combined with a convolutional neural network (CNN) and long-term memory (LSTM). The results demonstrated that the method achieved a prediction error of between 21.62% and 52.45% compared to other models. In addition, the model could provide important information for decision-making within air traffic management.

Similarly, Shi, Pan & Xu (2020) suggested a system based on ArcGIS 10.0 for the management of aerial trajectories through the LSTM network. The aim of this study was to provide ancillary decisions (or options) for air system operators. The results demonstrated that the system could predict flight paths with high accuracy and efficiency, supporting decision-making in the short term. Saâdaoui, Saadaoui & Rabbouch (2020) proposed a model for predicting traffic conditions in the US air network by decomposing time series and artificial neural networks (feedforward). The results were optimal since the model could generate accurate forecasts within acceptable time.

Lu et al. (2021) proposed the use of neural networks (NNs) to examine the effects of various transport networks on the heterogeneous spread of COVID-19 in China. They proposed a model called transport proximity deep neural network weighted regression (PDNNWR), which combined spatial heterogeneity for the spread of the pandemic, the relevance of transport proximity in human movement, and the accuracy of a deep neural network. The PDNNWR method achieved a more accurate prediction than the geographically weighted regression method. The results indicated that the spread of the virus through the air transport network was particularly high, even without any direct flight connections to the epicenter of the pandemic.

Finally, Choi & Kim (2021), proposed a model for air traffic management using artificial neural networks. The objective of this study was to predict the arrival and departure capacity of Atlanta’s Hartsfield–Jackson International Airport (ATL), using the multilayer perceptron (MLP), recurrent neural networks (RNN), and LSTM models. The results indicated that the artificial neural network approach is effective in predicting aircraft arrivals and departures.

### Traveling salesman problem

As an example of the traveling salesman problem (TSP), Wang, Yu & Liu (2018) developed an algorithm using the probability density distribution and the Bayesian formula based on the TSP. The aim of the investigation was to generate a rescue plan for air accidents. According to the results, the algorithm could generate a search plan by identifying the area and generating an optimal search path. In addition, the algorithm was 4% faster than the current strategies.

Duque et al. (2018) proposed an algorithm for the management of optimal routes that also used the TSP in addition to the ant colony optimization (ACO) strategy. The objective of this study was to determine the best flights for visiting certain cities. The results were acceptable since the algorithm generated optimal routes in a short time while also considering the cost of the trips.

Similarly, Muren et al. (2019) developed an air logistics model using incremental heuristic and mixed optimization algorithms. The results indicated that the proposed model performed better than exact and intelligent algorithms. In addition, the model generated optimal aircraft routing with approximately 99.5% accuracy within a short computational time (according to the number of nodes).

Marques, Russo & Roma (2019) developed a model using the TSP, which employed different heuristic and metaheuristic optimization algorithms. The goal of the model was to find the best schedule, route, and set of flights for any unrestricted flight request. The results were acceptable, and the model generated different routes with and prices that were up to 35% cheaper, allowing users to save time and money.

Alrasheed et al. (2019), proposed a model for the optimization of air transport services using the TSP and a local search algorithm. The main objective of the study was to find an optimal route that passed only once through each defined air terminal. The results facilitated the generation of low-cost air trajectories between airports in a short time.

In addition, Ahmad, Muklason & Nurkasanah (2020) recently developed an algorithm for the optimization of air routes using the TSP and a search algorithm called the hybrid tabu. The aim of this study was to find travel routes with the lowest possible costs and then compare them with the great deluge algorithm. Their algorithm was tested with several datasets (both small and medium). The results were encouraging because different optimal routes were generated with a 48.54% confidence level compared to the great deluge algorithm.

Pylyavskyy, Kheiri & Ahmed (2020), proposed a method for the optimization of flight connections based on the TSP through the reinforcement learning algorithm. The main objective of this study was to determine economical air routes between two airports. The results indicated that the proposed method could generate ideal routes while minimizing total travel costs. Tomanová & Holý (2020) proposed a heuristic method using an ant colony algorithm based on a modified TSP. The objective of the research was to determine optimal routes supported by the Hamiltonian cycle with minimal cost. The results were acceptable, and the method generated effective trajectories according to the parameters of cost, distance, and city per day in a short time.

Guevara & Penas (2020) developed the intelligent infectious diseases algorithm (IIDA), which could locate the sources of contagion and determine the survival rate of the COVID-19 virus using kernel density (KDE), ant colony optimization algorithms, and the TSP. The main goal of this study was to reduce the spread of the virus and decrease the number of infections. The algorithm was tested with data from obtained from New York State. The results were acceptable since the IIDA algorithm managed to determine areas with higher rates of infection and mortality and generate optimal routes of medical care within a reasonable processing time.

Finally, Bulbul & Kasimbeyli (2021) developed a hybrid method using Gasimov’s algorithm and the ant colony algorithm based on the TSP. The aim of the research was to generate optimal routing for aircraft maintenance. This method analyzed the traffic of air routes and maintenance effectively, generating optimal routes that did not violate any defined routes.

### Graph theory

Bae et al. (2018) proposed an algorithm to solve routing and flight scheduling problems using graph theory and the first-come-first-served algorithm. The results were optimal, with the algorithm solving iteration problems in routing by generating flights with multiple trajectories and aircraft speed profiles.

Along similar lines, Bhapkar, Mahalle & Dhotre (2020), analyzed the impact of the COVID-19 pandemic on the world by using the graph theory approach. The main objective of the study was to understand and graph the spread of COVID-19 infections. The results indicated different situations of contagion through geolocation. In addition, with a 1% growth rate of infections and an infection rate of 5, the infections doubled in just 15 days.

Small et al. (2020) proposed a model for analyzing the spread of COVID-19 in the cities of Australia and Perth. This model used graph theory to generate a network topology and the SIR model to determine the dynamic transmission of diseases. The results provided a useful prediction for the generation of control strategies and effective policy formulation to reduce the rate of infection. In addition, it was determined that strict compliance with isolation could decrease the probability of spreading COVID-19 by 80%.

Wijayanto & Ridho (2020) developed a model for managing future flight paths. Their model used graph theory for link prediction by mapping existing airports as vertices and flight paths as links. The method achieved an average prediction accuracy of 90%.

Neretin, Budkov & Ivanov (2020), analyzed the problem of implementing navigation routes in civil aviation using the A-star method of graph theory. The results were satisfactory since the proposed method provided four different optimization criteria considering wind conditions, no-fly zones, and aircraft performance.

Serafino (2020) developed a methodology using graph theory for determining the best trajectory in terms of fuel consumption, climatic conditions, and reduction of gas emissions. The results provided a trajectory with high precision for multi-objective routes in a short time.

Ekpenyong et al. (2020), presented a model called Spatio-GraphNet for tracing the people infected by COVID-19 using graph theory. The results were optimal, as the Spatio-GraphNet model generated real-time spatial information on sources of contagion and the predictive behavior of the spread of COVID-19. In addition, the use of graph theory facilitated tracking infected people effectively and promptly. Ivashchuk et al. (2021) proposed a method for analyzing Ukraine’s air traffic management using graph theory. The aim of the study was to examine the configuration of the flight path network. The results were acceptable since the method generated optimal flight paths that would help improve air navigation.

Gaeta, Loia & Orciuoli (2021), presented a method called 3WD for analyzing the spread of COVID-19. The 3WD method used graph theory and proximal three-way decision-making, and the data were provided by the Italian Department of Civil Protection. The results were encouraging, helping decision-making through contagion thresholds and critical infrastructure.

Finally, Isufaj, Koca & Piera (2021) conducted another study using graph theory for modeling air traffic, with the main objective of simplifying air traffic management. The results were encouraging, as method generated indicators that could measure several structural properties of air traffic, including edge density (graph size), strength (severity of interdependencies), clustering coefficient (proximity of each aircraft), and degree of proximity. In addition, the model provided a detailed view of how aircraft interactions can change over time.

### Markov chain

Tolić, Kleineberg & Antulov-Fantulin (2018), analyzed the time taken for the spread of pandemics using three different empirical networks. A framework for the simulation of Poisson’s SIR model was developed to find the shortest route by applying Markov chains that could identify epidemic spread times using Poisson’s mathematical model. The proposed framework could determine the estimated time of spread and detect possible sources of contagion.

Similarly, Ayhan, Costas & Samet (2018) proposed a novel system for air conflict management in long-range aircraft. This system used real data from 16 airports in Europe and the United States. The objective of the system was to analyze and detect air trajectories that violated protected areas using the Viterbi algorithm and Markov chains. The results proved efficient, and the system generated different alternative routes that did not violate the protected airspace.

Lin, Wei Zhang & Liu (2018) proposed a model to generate trajectory plans through machine learning and Markov chains. The objective of the study was to predict flight paths based on the relative movement between positions (*i.e*., speed, direction, and angular movement). The results demonstrated that the model could generate excellent solutions for predicting trajectories with high accuracy. Similarly, Delahaye, Chaimatanan & Mongeau (2019) developed a methodology for strategically managing the trajectories of 30,000 flights in Europe using a simulated annealing algorithm and Markov Chains (MC) with a Monte Carlo approach. The objective of the study was to plan the trajectories of large-scale aircraft. The results indicated that the methodology generated strategic planning that minimized the number of interactions between trajectories, helping to improve air management in the same period.

Kieckbusch et al. (2019) proposed a model for air traffic flow management that employed Markov chains and reinforcement learning. The aim was to ensure that the aircraft could take off and land safely, with minimal delays. The results were acceptable because the method generated optimal routes that considered different parameters, such as ground delays, decelerations enroute, and detours to less congested airports.

Rodríguez-Sanz et al. (2019) developed a methodology for predicting and evaluating the operational status of the air traffic system at Adolfo Suárez Madrid–Barajas Airport (LEMD) using a Bayesian network and the Markov chain (MC) with a Monte Carlo approach. The results were acceptable, minimizing congestion problems and air delays by 70%. In addition, the method helped decision-making in cases of operational uncertainty.

Sa, Santos & Clarke (2020) presented a methodology for planning airline fleets using Markov chains. The aim was to identify robust fleet plans that met the demands for long-term travel. Their methodology offered superior planning of air fleets with a greater flight frequency to capture a larger part of the market.

He et al. (2020), presented a model for improving the operational safety of the Shanghai airport airspace system using a Markov chain and set pair analysis (SPA). The method proved effective and feasible in accurately describing the characteristics of dynamic change. In addition, the method was simple and easy to operate. Similarly, Ikai, Miyagi & Sakai (2021) presented a method for planning air routes with multiple locations using a Markov chain. The results were adequate, generating optimal logistics planning for air trajectories. In addition, this method could be implemented for both air networks and vehicular traffic.

Guevara & Bonilla (2021) presented an algorithm for preventing the spread of infectious diseases on air routes and in airports from Wuhan in China (WHU) to different countries around the world. The authors applied Dijkstra’s algorithm, fuzzy logic, graphs, and the Markov chain. The proposed algorithm used information from airports, routes provided by flight connections, and COVID-19 spread data from Johns Hopkins University. As a result, the 25.23% airports could be identified as “high dispersion,” meaning that they should adopt more health and mobility controls. With regard to air routes, 30.80% were considered high risk (in terms of spreading the virus), leaving approximately 850,000 passengers infected with the virus in a month.

### Fuzzy logic

Fuzzy logic (FL) is widely applied to solve problems involving shortest routes, as exemplified by Savkin & Huang (2017) presented a method for generating optimal trajectories for military aircraft in harsh environments using an algorithm based on fuzzy logic. The results were acceptable since the method generated an optimal route while minimizing trajectory length and threat levels.

Volpe Lovato et al. (2018) presented a hybrid model for detecting and resolving conflicts on air traffic routes that used a genetic algorithm and fuzzy logic. The objective was to calculate the optimal actions in terms of changes in flight levels between the aircraft through a global and dynamic analysis. The results were encouraging, with the model being able to detect and eliminate longitudinal conflicts in advance and support decision-making effectively for tower controllers.

Similarly, Perçin (2018) proposed a method called VIKOR to evaluate and classify the performance and service of airlines in Turkey using fuzzy logic. The objective was to improve the competitive advantage over other countries, and the results could help decision-making for improving air operation management.

In the same area of fuzzy logic, Dudeja (2019) presented a study in which a modified fuzzy algorithm was applied to an empirical database. The main objective was to propose a novel strategy to reduce costs and time consumption using fuzzy rules to overcome the problem of the shortest path. In addition, the modified fuzzy algorithm was compared with different metaheuristic algorithms. The results demonstrated an improvement in coding efficiency, time consumption, and costs in dynamic path generation.

Kaleta & Skorupski (2019) proposed a model and implemented a hierarchical blurred inference system to determine the probability of air accidents. The objective of the study was to evaluate air traffic safety, the probability of air accidents, and the implementation of guidance procedures using controlled flight into the terrain. This was based on fuzzy logic and hierarchical fuzzy inference systems. The model considerably decreased the probability of air accidents and could be used commercially in small aerodromes.

Lovato et al. (2019), proposed a model for controlling conflicts on air traffic routes. The objective was to quantify the levels of longitudinal conflict between the aircraft and provide acceleration based on the level of conflict detected using fuzzy logic. The model could detect and eliminate longitudinal conflicts in advance and function well without compromising safety or violating existing air regulations.

Olive et al. (2020) developed a model for detecting events in aircraft trajectories using fuzzy logic. The objective was to conduct a large-scale analysis of the trajectories in the flight phase, flight plans, airspace, and operating procedures. The model provided highly valuable information for monitoring and situational decision-making.

Heydari, Omrani & Taghizadeh (2020) proposed a model for assessing the efficiency of Iran’s airlines. The goal was to design a network structure for airlines and calculate the efficiency score. The results suggested that the overall efficiency of airline operations is low. In addition, the method provided important information for improving air route operations. Mijovic, Kalic & Kuljanin (2021) developed a fuzzy logic model using metaheuristic algorithms to determine the market share of airlines on long-haul routes in the London airport system. The model significantly increased the efficiency of market share value prediction and could improve the operational performance of airlines.

Finally, Sudakov, Francisco & López De Hierro (2021), developed a method for planning air travel in Russia using fuzzy logic. The aim was to generate a diffuse air traffic matrix (origin–destination) between airports and classify them into potentially promising routes. The method solved the problems of transport route planning, aircraft choice, and the design of new airports. In addition, the model supported decision-making on improving air transport infrastructure.

Table 1 lists the studies analyzed in the literature review. This table highlights the current state of studies in this area, demonstrating extensive research related to route generation. In addition, Table 1 presents the algorithms and techniques employed in these studies with the symbol “ ${\checkmark}$”. The following criteria are grouped: evolutionary algorithms (EA), dynamic programming (DP), Dijkstra’s algorithm (DJK), neural networks (NN), graph theory (GT), Markov chain (MC), and fuzzy Logic (FL).

**Table 1:** Review of algorithms and optimal route techniques.

Related works	EA	DP	DJK	NN	TSP	GT	MC	FL
Savkin & Huang (2017)								$\checkmark$
Tolić, Kleineberg & Antulov-Fantulin (2018)							$\checkmark$	
Wang, Yu & Liu (2018)				$\checkmark$	$\checkmark$			
Ho-Huu et al. (2018)	$\checkmark$							
Kammoun & Rezg (2018)	$\checkmark$							
Wu et al. (2018)	$\checkmark$							
Azis et al. (2018)		$\checkmark$						
Prakash, Piplani & Desai (2018)		$\checkmark$						
Adacher & Flamini (2018)			$\checkmark$					
Gao et al. (2018)			$\checkmark$					
Xiao, Cai & Abbass (2018)			$\checkmark$					
Duque et al. (2018)					$\checkmark$			
Bae et al. (2018)						$\checkmark$		
Ayhan, Costas & Samet (2018)		$\checkmark$		$\checkmark$			$\checkmark$	
Lin, Wei Zhang & Liu (2018)				$\checkmark$			$\checkmark$	
Volpe Lovato et al. (2018)								$\checkmark$
Perçin (2018)								$\checkmark$
Brown, Hirabayshi & Wickramasinghe (2019)		$\checkmark$						
Kaleta & Skorupski (2019)								$\checkmark$
Dabachine, Bouikhalene & Balouki (2019)			$\checkmark$					
Alieksieiev (2019)	$\checkmark$					$\checkmark$		
Yu & Wang (2019)		$\checkmark$						
Tian et al. (2019)		$\checkmark$						
Petrov (2019)			$\checkmark$					
Lin, Wei Zhang & Liu (2019)				$\checkmark$				
Liu et al. (2019)				$\checkmark$				
Wang et al. (2019)							$\checkmark$	
Han et al. (2019)				$\checkmark$				
Muren et al. (2019)					$\checkmark$			
Marques, Russo & Roma (2019)	$\checkmark$				$\checkmark$			
Alrasheed et al. (2019)					$\checkmark$			
Rodríguez-Sanz et al. (2019)							$\checkmark$	
Delahaye, Chaimatanan & Mongeau (2019)							$\checkmark$	
Kieckbusch et al. (2019)				$\checkmark$			$\checkmark$	
Dudeja (2019)								$\checkmark$
Lovato et al. (2019)		$\checkmark$						$\checkmark$
Ntakolia, Caceres & Coletsos (2020)		$\checkmark$						
Guevara & Penas (2020)					$\checkmark$			
Yanzhe & Bingxiang (2020)	$\checkmark$							
Turky et al. (2020)	$\checkmark$							
Cai et al. (2020)	$\checkmark$							
Weerasinghe et al. (2020)	$\checkmark$							
Chen & Solak (2020)		$\checkmark$						
Yi, Tong & LiuXi (2020)		$\checkmark$						
Weiszer, Burke & Chen (2020)			$\checkmark$					
Dudi et al. (2020)			$\checkmark$					
Jazzar et al. (2020)			$\checkmark$			$\checkmark$		
Zhao et al. (2020)			$\checkmark$					
Wang et al. (2020)				$\checkmark$		$\checkmark$		
Ma & Tian (2020)				$\checkmark$				
Shi, Pan & Xu (2020)				$\checkmark$				
Saâdaoui, Saadaoui & Rabbouch (2020)				$\checkmark$				
Ahmad, Muklason & Nurkasanah (2020)					$\checkmark$			
Pylyavskyy, Kheiri & Ahmed (2020)				$\checkmark$	$\checkmark$			
Tomanová & Holý (2020)					$\checkmark$			
Bhapkar, Mahalle & Dhotre (2020)						$\checkmark$		
Small et al. (2020)						$\checkmark$		
Ekpenyong et al. (2020)						$\checkmark$		
Wijayanto & Ridho (2020)				$\checkmark$		$\checkmark$		
Neretin, Budkov & Ivanov (2020)						$\checkmark$		
Serafino (2020)						$\checkmark$		
He et al. (2020)							$\checkmark$	
Sa, Santos & Clarke (2020)							$\checkmark$	
Olive et al. (2020)								$\checkmark$
Heydari, Omrani & Taghizadeh (2020)								$\checkmark$
Fijar Awalivian, Suyanto & Sa’Adah (2021)	$\checkmark$							
Cai, Ang & Alam (2021)	$\checkmark$							
Ahmed, Bousson & Coelho (2021)		$\checkmark$						
de Campos, Vieira & Munari (2021)		$\checkmark$						
Maristany de las Casas, Sedeño-Noda & Borndörfer (2021)			$\checkmark$					
Lu et al. (2021)				$\checkmark$				
Choi & Kim (2021)				$\checkmark$				
Bulbul & Kasimbeyli (2021)					$\checkmark$			
Gaeta, Loia & Orciuoli (2021)						$\checkmark$		
Ivashchuk et al. (2021)						$\checkmark$		
Isufaj, Koca & Piera (2021)						$\checkmark$		
Ikai, Miyagi & Sakai (2021)							$\checkmark$	
Sudakov, Francisco & López De Hierro (2021)								$\checkmark$
Mijovic, Kalic & Kuljanin (2021)								$\checkmark$
Guevara & Bonilla (2021)			$\checkmark$			$\checkmark$	$\checkmark$	$\checkmark$

The main objective of these works was to find the shortest route through some methodologies and then compare them (or establish rules) to improve the air transport system in a pandemic situation. Many of these studies ended in the analysis phase and focused mainly on improving the air transport system. However, they did not generate routes for the prevention and spread of diseases. The main difference between the studies reported in the literature analysis and the research presented here is that their objective was to determine different optimal routes between airports.

In Table 1, it is possible to identify works that have simultaneously applied one or more algorithms and techniques. The most used methods and algorithms are neural networks (14 studies) and graph theory (14 studies), and the rest of the algorithms have been applied 11 times each. These outcomes indicate that the neural networks and graph theory have been extensively analyzed with optimal research results. For this reason, we decided to apply graph theory (GT) as the fundamental base of our proposed method and Dijkstra’s algorithm (DJK) to offer another approach to the research area.

## Materials

In this section, details of the materials used for the development of this study are presented (databases of airports of the international air network). In addition, we used the database of COVID-19 infections since the beginning of the pandemic in 2020, and its space-time (geographic location and date the infection was reported) spread until 2022.

### Airport database IATA

The airport database includes information from all international air terminals worldwide. This dataset was compiled by the International Air Transport Association (IATA) (Aviation & the environment, 2019). The dataset comprises 26,914 records with the following eight variables: IATA code, latitude, longitude, number of routes, country, continent, origin, and destination of the air terminal (Table 2).

**Table 2:** Airport database (Aviation & the environment, 2019).

Name	Type	Precision	Example
IATA code	Text	Three characters	HEA
Latitude	Numerical	Whole with five decimals	34.21263
Longitude	Numerical	Whole with five decimals	62.22651
Number of routes	Numerical	Whole	1
Country	Text	Characters	Afghanistan
Continent	Text	Characters	Asia
IATA origin	Text	Three characters	HEA
IATA destination	Text	Three characters	MHD

The distribution of existing international airports worldwide, classified by continent, where Asia (26.75%), Europe (34.85%), America (21.90%), Oceania (3.90%), and Africa (12.57%). The most significant number of international airport terminals are concentrated in Asia and Europe, which constitute 61.60% of the total number of international airports.

The top 10 countries with the most significant number of international airports are China, which has the highest number of international airports (75), followed by the United States (71), Russia (65), France (44), Italy (32), United Kingdom (32), Germany (31), Japan (31), Mexico (30) and Spain (30).

The top 10 countries with the highest number of international air routes are: The United Kingdom has 1,776 routes, followed by Germany with 1,503 routes, the United States with 1,417 routes, Spain with 1,410 routes, Italy with 1,266 routes, France with 1,231 routes, China with 990 routes, Greece 848 routes and Turkey 670 routes.

**Figure 1:**
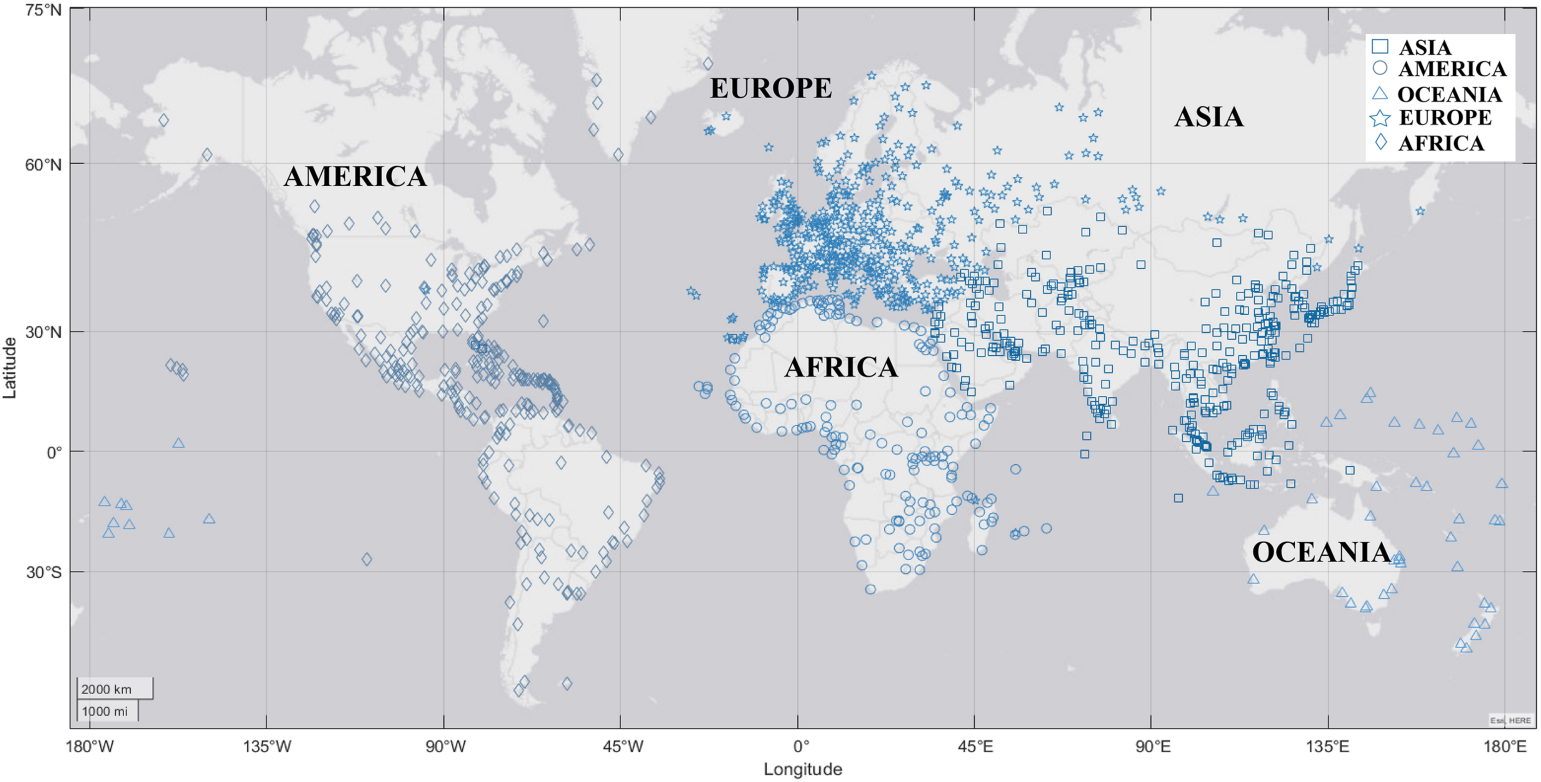
Geospatial location of international air terminals.

Figure 1 shows the geospatial location (latitude and longitude) of the air terminals of all airports in the world, classified by continent. This information allowed us to identify the distance between international air terminals as well as existing connections.

With this information, we could describe the connections between each of the international terminals, as well as their distance and exact position.

### COVID-19 dataset

This data repository collects information on the global spread of COVID-19 and was collected by the Center for Systems Science and Engineering at Johns Hopkins University (Center for Systems Science & Engineering (CSSE) at Johns Hopkins University (JHU), 2023). The dataset has 152,736 records collected between February 1, 2020, and January 5, 2022. In addition, it has seven variables: country, date, confirmed cases, deaths, recovered, active cases, and region (Table 3).

**Table 3:** COVID-19 dataset (Center for Systems Science & Engineering (CSSE) at Johns Hopkins University (JHU), 2023).

Name	Type	Precision	Example
Country	Text	Characters	China
Date	Date	Date	22/01/2020
Confirmed	Numerical	Whole	444
Deaths	Numerical	Whole	17
Recovered	Numerical	Whole	28
Active	Numerical	Whole	399
Region	Text	Characters	Western Pacific

The number of COVID-19 infections is categorized by continent, where America is the one with the highest number of positive cases (109,766.438 positive cases), followed by Europe (92,815.435 positive cases), Asia (85,568.026 positive cases), Africa (10,039.737 positive cases), Oceania (85,568.026 positive cases).

The top 10 countries with the highest infection rates are The United States (58,805,186 cases), followed by India (35,109,286), Brazil (22,351,104), United Kingdom (14,015,065), France (10,921,757), Russia (10,601,300), Turkey (9,718,861), Germany (7,360,556), Italy (6,975,465), and Spain (6,922,466).

**Figure 2:**
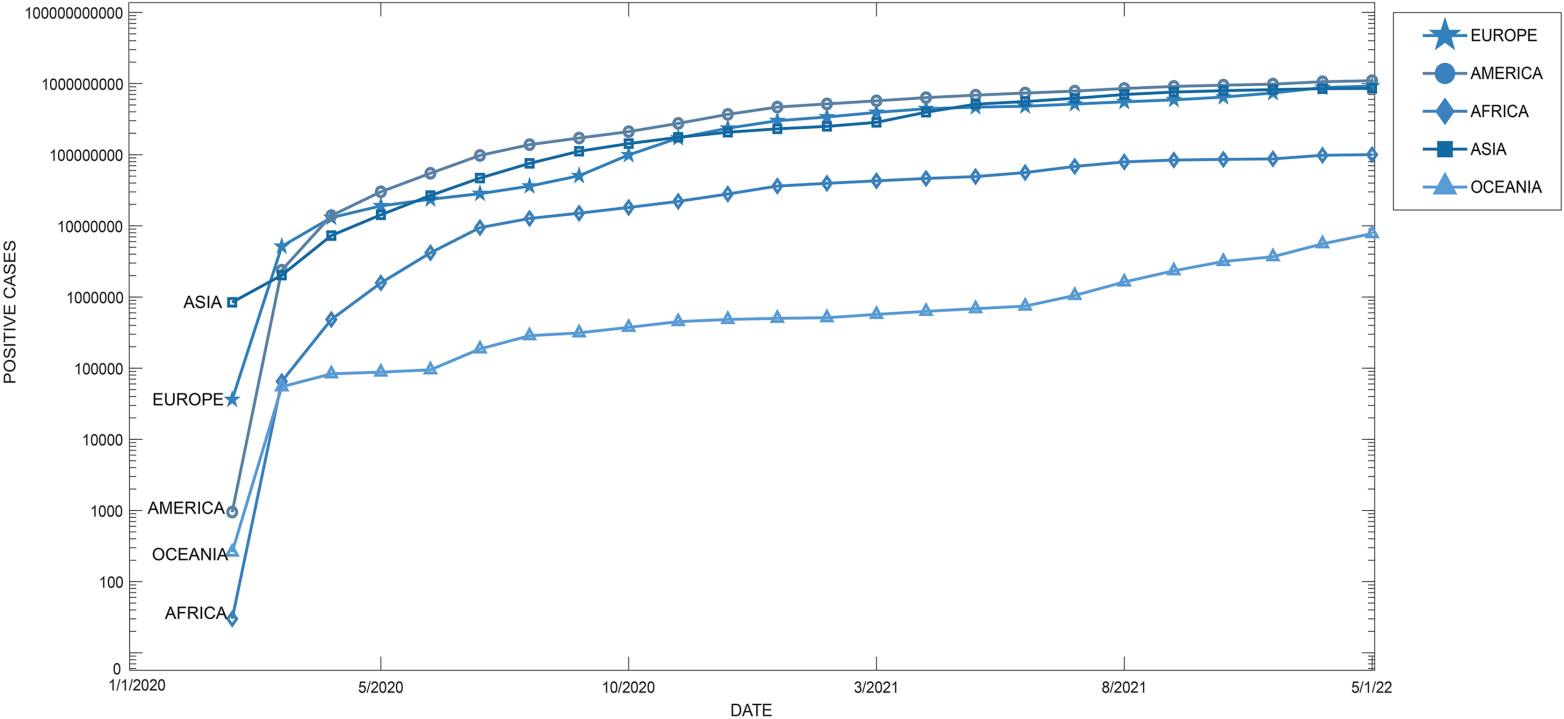
Timeline of increase in COVID-19 infections by continent.

Figure 2 presents a chronological diagram of COVID-19 infections classified by continent from February 2020 (880,480 confirmed cases) to January 5, 2022 (2,981,694,750 confirmed cases).

**Figure 3:**
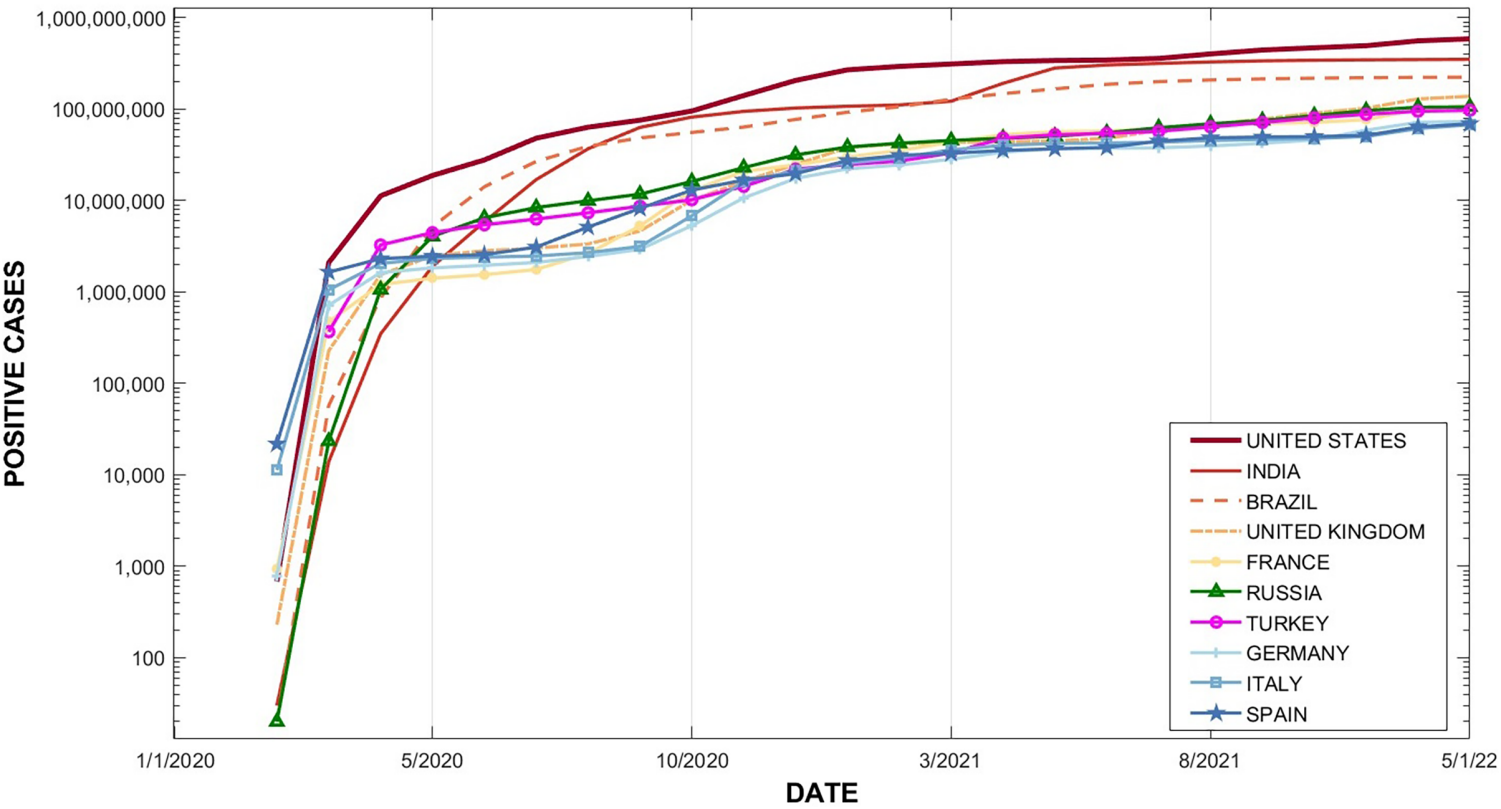
Timeline of top 10 countries, increases in COVID-19 infections.

Figure 3 presents the timeline of COVID-19 spread in the top 10 countries with the highest number of infections, from January 1, 2020, to January 05, 2022.

Table 4 presents the temporal chronology of the spread of COVID-19 for each country. This information is grouped by date (categories every 15 days) from January 15 to March 15, 2020, in countries where cases of the disease have been identified.

**Table 4:** Timeline of COVID-19 spread by country.

Start date	End date	Group	Infected countries
15/01/2020	31/01/2020	Group 1	China, Japan, South Korea, Taiwan, Thailand, US, Singapore, Vietnam, France, Malaysia, Nepal, Australia, Canada, Cambodia, Germany, Sri Lanka, Finland, United Arab Emirates, India, Philippines, Italy, Russia, Sweden, United Kingdom.
01/02/2020	15/02/2020	Group 2	Spain, Belgium, Egypt
16/02/2020	29/02/2020	Group 3	Iran, Israel, Lebanon, Chile, Afghanistan, Bahrain, Iraq, Kuwait, Oman, Algeria, Austria, Croatia, Pakistan, Switzerland, Brazil, Georgia, Greece, North Macedonia, Norway, Romania, Denmark, Estonia, Netherlands, San Marino, Belarus, Iceland, Lithuania, Mexico, New Zealand, Nigeria, Ireland, Luxembourg, Monaco, Qatar.
01/03/2020	15/03/2020	Group 4	Armenia, Azerbaijan, Czechia, Dominican Republic, Ecuador, Andorra, Indonesia, Latvia, Morocco, Portugal, Saudi Arabia, Senegal, Argentina, Jordan, Ukraine, Hungary, Liechtenstein, Poland, Tunisia, Bosnia and Herzegovina, Slovenia, South Africa, West Bank and Gaza, Bhutan, Cameroon, Colombia, Costa Rica, Holy See, Peru, Serbia, Slovakia, Togo, Malta, Bangladesh, Bulgaria, Maldives, Moldova, Paraguay, Albania, Brunei, Cyprus, Burkina Faso, Mongolia, Panama, Bolivia, Congo (Kinshasa), Cote d’Ivoire, Honduras, Jamaica, Turkey, Cuba, Guyana, Antigua and Barbuda, Ethiopia, Guinea, Kazakhstan, Kenya, Sudan, Uruguay, Eswatini, Gabon, Ghana, Guatemala, Mauritania, Namibia, Rwanda, Saint Lucia, Saint Vincent and the Grenadines, Seychelles, Suriname, Trinidad and Tobago, Venezuela, Central African Republic, Congo (Brazzaville), Equatorial Guinea, Uzbekistan.
16/03/2020	31/03/2020	Group 5	Bahamas, Benin, Greenland, Liberia, Somalia, Tanzania, Barbados, Gambia, Montenegro, Kyrgyzstan, Mauritius, Zambia, Chad, El Salvador, Fiji, Nicaragua, Angola, Cape Verde, Haiti, Madagascar, Niger, Papua New Guinea, Zimbabwe, Eritrea, Uganda, Dominica, Grenada, Mozambique, Syria, Timor-Leste, Belize, Laos, Libya, Guinea-Bissau, Mali, Saint Kitts and Nevis, Kosovo, Burma, Botswana, Burundi, Sierra Leone.
01/04/2020	15/03/2020	Group 6	Malawi, South Sudan, Western Sahara, Sao Tome and Principe, Yemen.
16/04/2020	30/04/2020	Group 7	Comoros, Tajikistan.
01/05/2020	15/05/2020	Group 8	Lesotho.

## Covid-19 spread analysis

This section, presents an analysis of the spread of the COVID-19 virus, where the network of air terminals was designed by applying graph theory. Subsequently, multiple route simulations were carried out by applying Dijkstra’s algorithm to identify the terminals where the routes of spread of the virus were concentrated. These were compared with chronological data of the infections.

### Application of graph theory to air terminals

This section describes the creation of an interconnected network of international air terminals (airports) by applying graph theory (Ekpenyong et al., 2020). The air terminal graph is based on the information obtained from the IATA database (described in section), as follows (Eq. (1)):

(1) $$G = (V_{i},V_{f}, d)$$

Here $V_{i}(latitude_{Vi}, longitude_{Vi})$ is the initial vertex and $V_{f}(latitude_{Vf}, longitude_{Vf})$ is the final vertex. Each of these vertices is defined by the latitude and longitude, which constitute the geographic location of each terminal. The distance d between each of the vertices $V_{i}$ and $V_{f}$ is calculated by applying the Euclidian distance of Eq. (2) (Behrens et al., 2018).

(2) $$d = \sqrt{ (latitud_{Vf} - latitud_{Vi})^2 + (longitud_{Vf} -longitud_{Vi})^2}$$

Graph *G* has 1,297 vertices (international airports) and 26,914 distances between the vertices (routes between the terminals).

Table 5 presents an example of the application of Eqs. (1) and (2) to create graph *G* from WHU to eight different airports around the world.

**Table 5:** Examples of vertices *V* and distances d of graph *G*, with spatial information of eight international airports.

Origin $V_i$		Destiny $V_f$		
Airport	Latitude/Longitude	Airport	Latitude/Longitude	Distance $d$
Wuhan Tianhe International Airport (WUH)	30.7774638/114.2119	John F. Kennedy International Airport (JFK) United States/America	40.6420923/−73.77775	188.24837
		Istanbul Airport (IST) Turkey/Europe	41.2610594/28.744115	86.10842
		San Francisco International Airport (SFO) United States/America	37.6218225/−122.37908	236.69004
		Mandalay International Airport (MDL) Myanmar/Asia	21.7059633/95.971117	20.37206
		Dubai International Airport (DXB) United Arab Emirates/Asia	25.2534848/55.365200	59.10547
		London Heathrow Airport (LHR) United Kingdom/Europe	51.4695942/−0.454080	116.51810
		Kansai International Airport (KIX) Japan/Asia	34.4318901/135.23033	21.33368
		Singapore International Airport (SIN) Singapore/Asia	1.3642523/103.9916	31.13826

**Figure 4:**
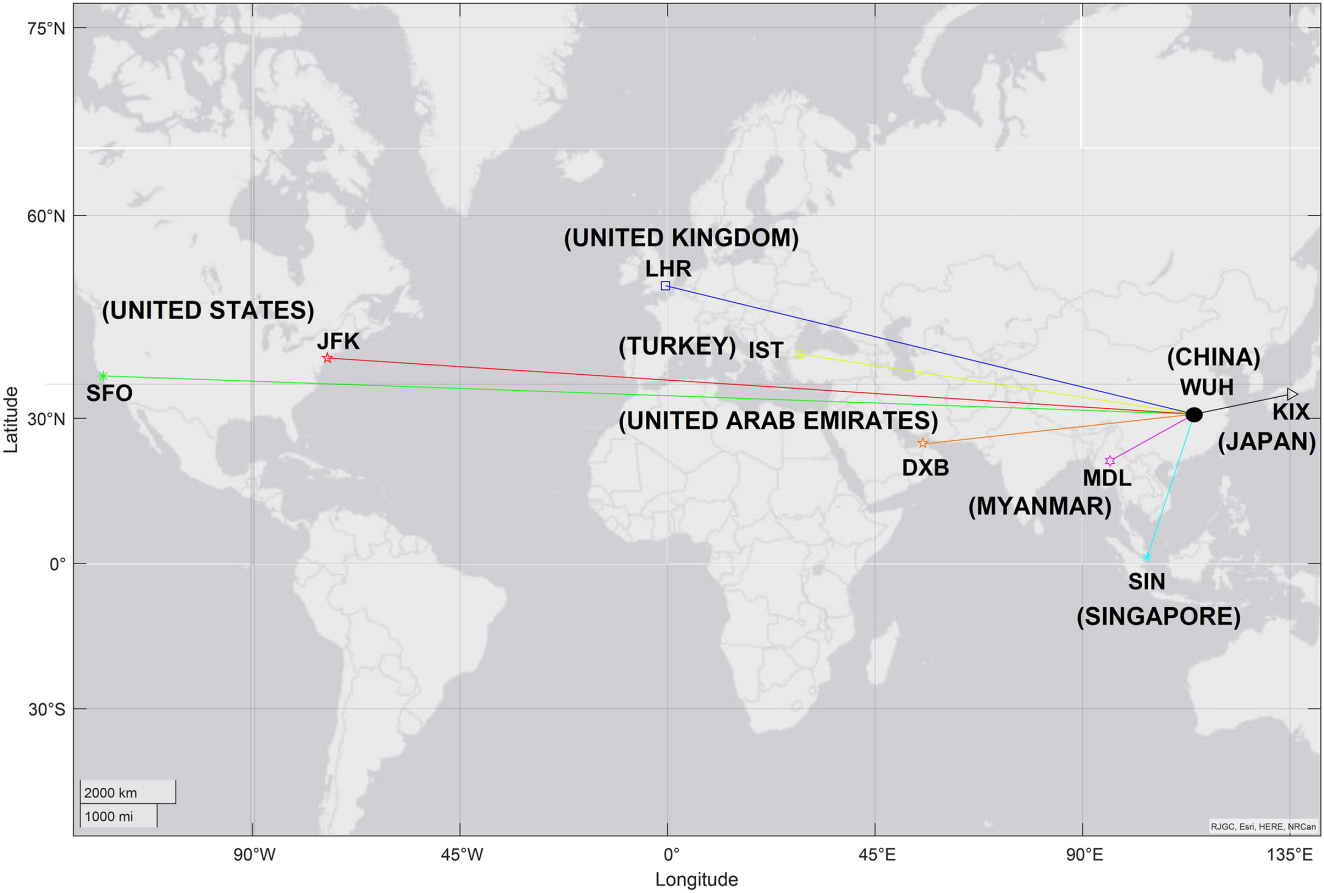
Geospatial map of graph *G*, from WHU to eight different terminals.

Figure 4 presents an example of graph *G* with international air terminals, where each line represents a distance $d$ (route) and each terminal represents a vertex *V*, from WHU to eight different airports in the continents of America, Europe, Asia, and Oceania.

### Application of Dijkstra’s algorithm for route generation

In this section, the generation of routes by applying Dijkstra’s algorithm (Bae et al., 2018; Baeza, Ihle & Ortiz, 2017) is described, from the airport of origin ( $V_i$) to the destination airport ( $V_f$). Algorithm 1 (Table 6) describes the pseudo-code for the generation of possible COVID-19 propagation routes on the international airport network, identifying the most crowded terminals and the most common air routes.

**Table 6:** Algorithm 1 Dijkstra’s algorithm pseudo code.

Function DIJKSTRA (Vi,G)	Function initialization vertexv in G
	*We go through each of the vertices of the Graph*
$dist [] \leftarrow 0$	*Vertex to vertex distance variable initialization*
$prev[] \leftarrow 0$	*Path Vertex List Variable Initialization*
**If** $V = V_i$ **then**	*We verify that the vertex is not different from the initial vertex*
$V \leftarrow AddPriority(Q)$	*We create a Q priority queue*
**While** $Q \neq \emptyset$ **do**	*We compare Q is not void*
$U \leftarrow$ Extract from *Q*	*We delete an item from Q that has already been visited*
Unvisited_neighbour(*V*) of *U*	*Let’s go through the unvisited vertex*
$temperoryDist \leftarrow dist[U] + edgeWeight_{(U,V)}$	*We assign a temporal distance*
**If** $temperoryDist \leftarrow dist[V]$ **then**	*We check if the time distance is less*
$dist[V] \leftarrow temperoryDist$	*We add to the distance vector*
$prev[V] \leftarrow U$	*We add to the variable the list of path vertices*
**End If**	
**End While**	
**End If**	
return dist[] prev[]	*We return the distance vector and the list of vertices of the optimal*
**End Function**	

These simulations were performed from the terminal of the city with the first reported COVID-19 case in WUH ( $V_{WUH}$) to 10 terminals in five continents, as presented in Table 7.

**Table 7:** International air terminals for the simulation of COVID-19 spread by applying Dijkstra’s algorithm.

Origin $V_i$		Destiny $V_f$	
Airport	Country/ Continent	Airport	Country/Continent
Wuhan-Tianhe International Airport (WUH)	China/Asia	Schiphol Amsterdam Airport (AMS)	Netherlands/Europe
		Frankfurt am Main Airport (FRA)	Germany/Europe
		Istanbul Airport (IST)	Turkey/Asia
		Dubai International Airport (DXB)	United Arab Emirates/Asia
		Mohammed V International Airport (CMN)	Morocco/Asia
		Bole Addis Ababa International Airport (ADD)	Ethiopia/Africa
		Toronto Pearson International Airport (YYZ)	Canada/America
		Jose Joaquin de Olmedo International Airport (GYE)	Ecuador/America
		Sydney Airport (SYD)	Australia/Oceania
		Auckland International Airport (AKL)	New Zealand/Oceania

For the simulation of propagation routes, the Determination of Airport Routes and Prevention of Disease Spread (DetARPDS) algorithm, as defined by Algorithm 2 (Table 8), was developed. This algorithm first generates an optimal path of graph *G* by applying Dijkstra’s Algorithm 1 (Table 6) from the start vertex $V_{WUH}$ to the destination vertex $V_f$ with the shortest distance. The resulting optimal path contains a list of connections between vertices $V_i$ and $V_f$, which will sequentially eliminate existing intermediate connections to determine possible propagation routes. This procedure is repeated until there is no connection between the start vertex $V_i$ and the destination vertex $V_f$. Subsequently, this is compared with the COVID-19 spread data in Table 4 to determine the routes that comply with the temporal chronology of the contagion. Finally, a set of resulting routes is obtained, wich determines the likeliest routes of the contagion from the origin to each of the selected destinations.

**Table 8:** Algorithm 2 pseudocode of DetARPDS.

Require: lataDataBase	
Require: categorialCovidDataBase	
Ensure: Vo	Origin airport
Ensure: Vf	Destination airport
$G \leftarrow []$	Graph of international airport network
$M \leftarrow []$	Matrix of routes from Vo to Vf
$R \leftarrow []$	Routes obtained for the algorithm
lengthG - length(IataDataBase)	Quantity of airports in lataDataBase
**For** $j \leftarrow 1$ **to** $lengthG - 1$ **do**	Vertex creation
$latitude_j \leftarrow lataDataBase_j$ (“*Latitude*”)	
$longitude_j \leftarrow lataDataBase_j$ (“*Longitude*”)	
$codeIATA_{j+1} \leftarrow lataDataBase_{j+1}$ (“*IATA − origin*”)	
$V_i(latitude_j, longitud_j, codeIATA_j)$	
$latitude_{j+1} \leftarrow lataDataBase_{j+1}$ (“*latitude*”)	
$longitude_{j+1} \leftarrow lataDataBase_{j+1}$ (“*Longitude*”)	
$codeIATA_{j+1} \leftarrow lataDataBase_{j+1}$ (“*IATA − destiny*”)	
$V_{j+1}(latitude_{j+1}, longitude_{j+1}, codeIATA_{j+ 1})$	
$d{j,j+1} \leftarrow EuclideanDistance(V_j, V_{j+1})$	Distance calculation
$G \leftarrow AddVertex(V_j,V_{j+1},d_{j,j+1})$	Add vertices into Graph G
**End For**	
$counter \leftarrow 0$	
**while** condition ==true **do**	
$counter \leftarrow counter+1$	
$route \leftarrow Dijkstra(V_i, V_f)$	
**if** route == 0 **then**	
$condition \leftarrow false$	
**Else**	
$M[counter] \leftarrow route$	
$G_j \leftarrow DeleteVertex(G, V_i)$	Delete vertex intermediate 1 of graph G
**End if**	
**End While**,	
$lenghtM \leftarrow length(M)$	Matrix M Compared to categorialCovidDataBase
**For** $k \leftarrow 1$ **to** $lenghtM$ **do**	
$R[k] \leftarrow CompTempPropCovid(M[k],categorialCovidDataBase)$	
**End for**	
Print $R[k]$	Routes obtained for the algorithm

## Results and discussion

To perform the simulations, we worked with 26.915 records from the IATA database and 49.069 records from the COVID-19 dataset. Testing was conducted using MATLAB R2020b version 9.0 on an INTEL Core i7 (6th Gen) CPU with 12.0 GB of RAM running on Windows 10 64-bit OS and a 2.0 GB NVIDIA GPU (GTX-950).

The simulation results are presented in Table 9, which details the number of routes generated, the maximum number of connections between terminals, and the most visited terminals from the airport of origin $V_{WHU}$ to the 10 destination terminals $V_f$.

**Table 9:** Results of simulations applying the DetARPDS algorithm.

Origin $V_i$	Destiny $V_f$	Routes generated	Connections	Terminals
$V_{WUH}$	$V_{AMS}$	3874	8	
				T1) IST-814 T6) HKG-209 T2) DXB-769 T7) SVO-137 T3) LGW-648 T8) BGY-114 T4) JFK-298 T9) BKK-114 T5) LHR-265 T10) ICN-112
	$V_{FRA}$	4625	6	
				T1) IST-1021 T6) LHR-285 T2) DXB-716 T7) HKG-190 T3) LGW-571 T8) DUS-139 T4) SVO-542 T9) TPE-133 T5) JFK-343 T10) AMS-127
	$V_{V_{IST}}$	5467	6	
				T1) LGW-1898 T6) SZG-176 T2) DXB-1030 T7) HKG-146 T3) SVO-879 T8) TPE-141 T4) LHR-358 T9) CDG-139 T5) JFK-318 T10) AMS-138
	$V_{V_{DXB}}$	6588	7	
				T1) IST-2195 T6) JFK-254
				T2) LGW-1543 T7) CDG-226 T3) SVO-964 T8) ZRH-199 T4) LHR-313 T9) LCA-191 T5) HKG-298T10) MAD-190
	$V_{V_{CMN}}$	5828	7	
				T1) IST-1831 T6) MAD-301 T2) LGW-1284 T7) FRA-239 T3) DXB-922 T8) HKG-230 T4) SVO-821 T9) AMS-214 T5) AGP-494 T10) CDG-205
	$V_{ADD}$	5787	6	
				T1) IST-2246 T6) ATH-578 T2) DXB-842 T7) MRS-510 T3) LGW-816 T8) VIE-496 T4) SVO-521 T9) GVA-357 T5) CDG-663 T10) TLV-343
	$V_{YYZ}$	6138	6	
				T1) IST-2090 T6) MAN-371 T2) LGW-982 T7) FRA-318 T3) DXB-934 T8) CDG-314 T4) SVO-784 T9) MAD-221 T5) OPO-444 T10) VIE-240
	$V_{GYE}$	3607	8	
				T1) IST-1928 T6) LGW-345 T2) AMS-1480 T7) PTY-158 T3) MAD-1374 T8) CUN-145 T4) SVO-423 T9) JFK-129 T5) DXB-416 T10) LTN-123
	$V_{SYD}$	1937	8	
				T1) IST-558 T6) JNB-115 T2) IAH-558 T7) BKK-97 T3) DXB-361 T8) DEL-90 T4) DOH-344 T9) TPE-90 T5) HKG-176 T10) PVE-85
	$V_{AKL}$	1609	8	
				T1) IST-479 T6) HKG-148 T2) DOH-350 T7) DEL-126 T3) IAH-283 T8) PVG-89 T4) DXB-282 T9) PEK-79 T5) ORD-192 T10) LAX-75
	Mean		7	

**Figure 5:**
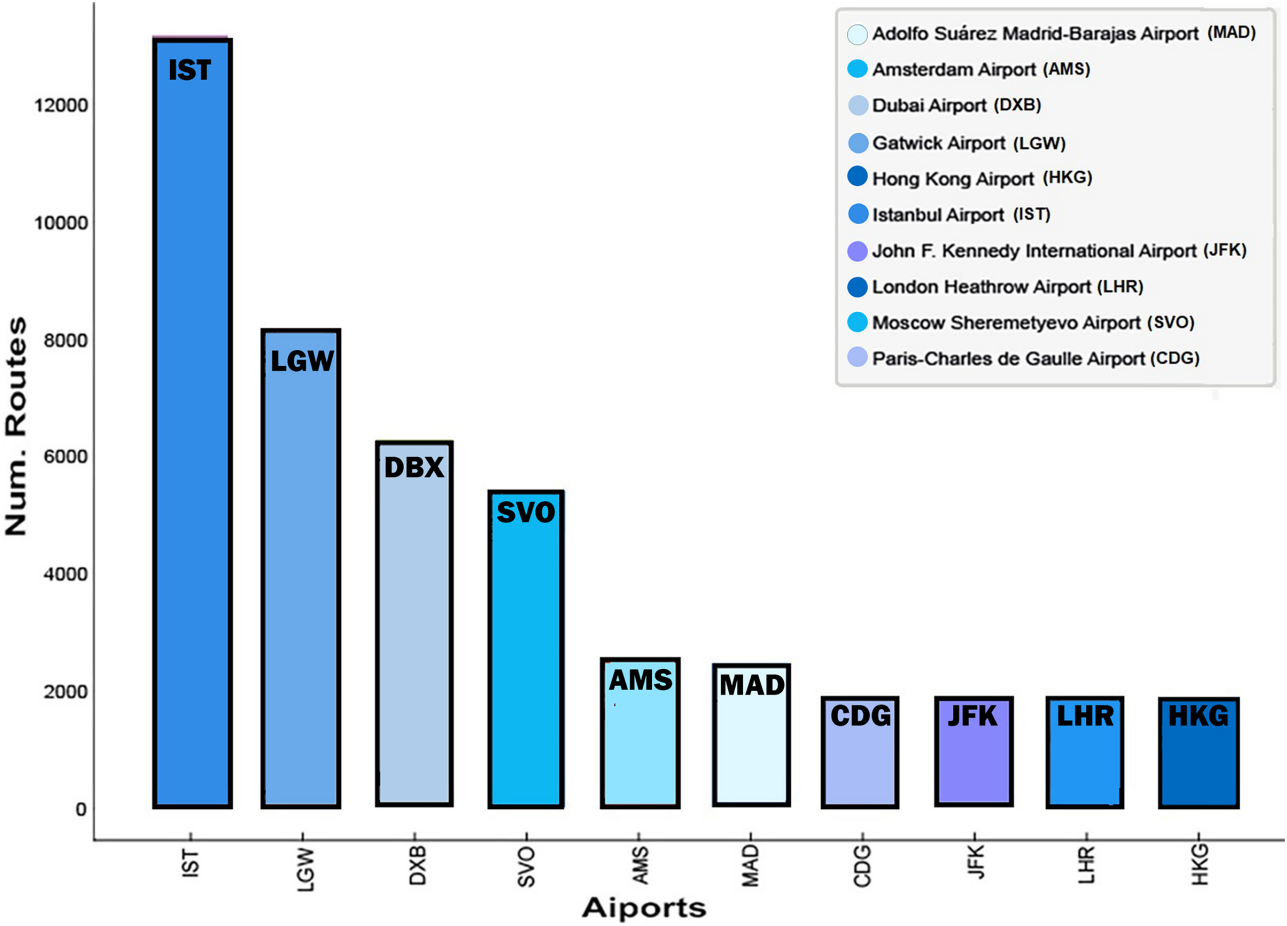
Busiest airports in the simulation by applying the DetARPDS algorithm.

As shown in the Table 9, the simulation of $V_{WUH }$ − $V_{YYZ}$ has the most routes generated (6.587). Figure 5 displays the 10 airports with the highest concurrence of simulated routes. Here, Istanbul Airport (IST) is the busiest (13.184 routes), followed by London-Gatwick Airport (LGW) (8.087 routes), Dubai International Airport (DXB) (6.295 routes), Moscow International Airport (SVO) (5.289 routes), Madrid–Barajas, Adolfo Suárez Airport (MAD) (2.116 routes), Amsterdam Schiphol Airport (AMS) (1.959 routes), Paris-Charles de Gaulle Airport (AMS) (1.959 routes), Hong Kong International Airport (HKG) (1.397 routes), John F. Kennedy International Airport (JFK) (1.342 routes), and London Heathrow Airport (LHR) (1.221 routes).

The busiest international air terminals for each continent were also determined, as shown in Table 10.

**Table 10:** Most visited international air terminals based on simulations of 10 destinations grouped by continent.

Continent	Country	Airport
Africa	Camerun	Douala International Airport (DLA)
Africa	Nigeria	Murtala MuhammadInternational Airport (LOS)
Africa	Ethiopia	Bole Addis AbabaInternational Airport (ADD)
Africa	Egypt	Cairo International Airport (CAI)
Africa	South Africa	O. R. Tambo International Airport (JNB)
America	United States	John F. Kennedy International Airport (JFK)
America	Panama	Tocumen InternationalAirport (PTY)
America	Colombia	El Dorado International Airport (BOG)
Asia	United Arab Emirates	Dubai InternationalAirport (DXB)
Asia	Hong Kong	Hong Kong InternationalAirport (HKG)
Asia	Thailand	Suvarnabhumi InternationalAirport (BKK)
Asia	Kazakhstan	Almaty InternationalAirport (ALA)
Asia	Sri Lanka	Bandaranaike InternationalAirport (CMB)
Europe	Turkey	Istanbul Airport (IST)
Europe	United Kingdom	Gatwick Airport (LGW)
Europe	Netherlands	Amsterdam Airport (AMS)
Europe	Spain	Adolfo Suarez Madrid–Barajas (MAD)
Oceania	Australia	Sydney Airport (SYD)

**Figure 6:**
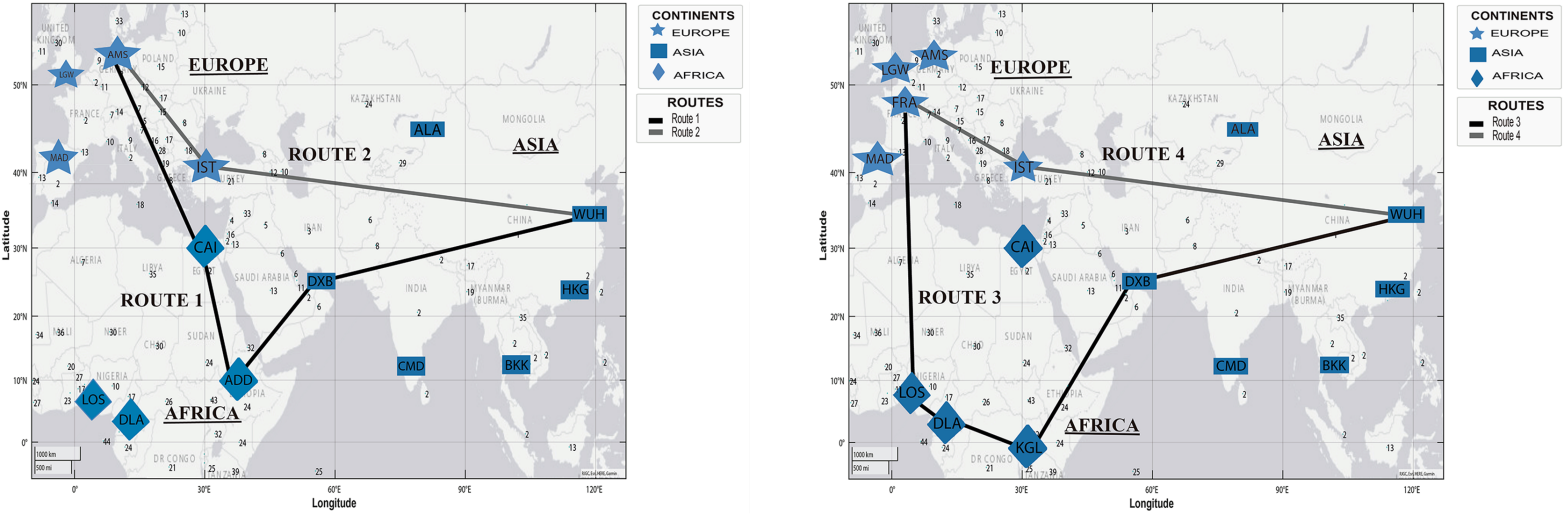
Simulation of COVID-19 spread routes from WHU to Istanbul (Turkey) and Frankfurt (Germany) by applying the DetARPDS algorithm.

Figure 6 shows the two routes simulated with the proposed DetARPDS algorithm from *WHU* to Amsterdam (Netherlands) and Frankfurt (Germany). In each of the simulations, two routes based on the chronology of the temporal spread of COVID-19 are presented in Table 4.

Route 1: WUH–DXB–ADD–CAI–AMS

- (WUH) WHU-Tianhe International Airport (Group 1)
- (DBX) Dubai International Airport (Group 1)
- (ADD) Bole Addis Ababa International Airport (Group 4)
- (CAI) Cairo International Airport (Group 2)
- (AMS) Amsterdam Airport (Group 3)

Route 2: WUH–IST–AMS

- (WUH) WHU-Tianhe International Airport (Group 1)
- (IST) Istanbul Airport (Group 4)
- (AMS) Amsterdam Airport (Group 3)

Route 3: WUH–DXB–KGL–DLA–LOS–FRA

- (WUH) WHU-Tianhe International Airport (Group 1)
- (DXB) Dubai International Airport (Group 1)
- (KGL) Kigali International Airport (Group 4)
- (DLA) Douala International Airport (Group 4)
- (LOS) Murtala Muhammad International Airport (Group 3)
- (FRA) Frankfurt Airport (Grupo 1)

Route 4: WUH–IST–FRA

- (WUH) WHU-Tianhe International Airport (Group 1)
- (IST) Istanbul Airport (Group 4)
- (FRA) Frankfurt Airport (Group 1)

**Figure 7:**
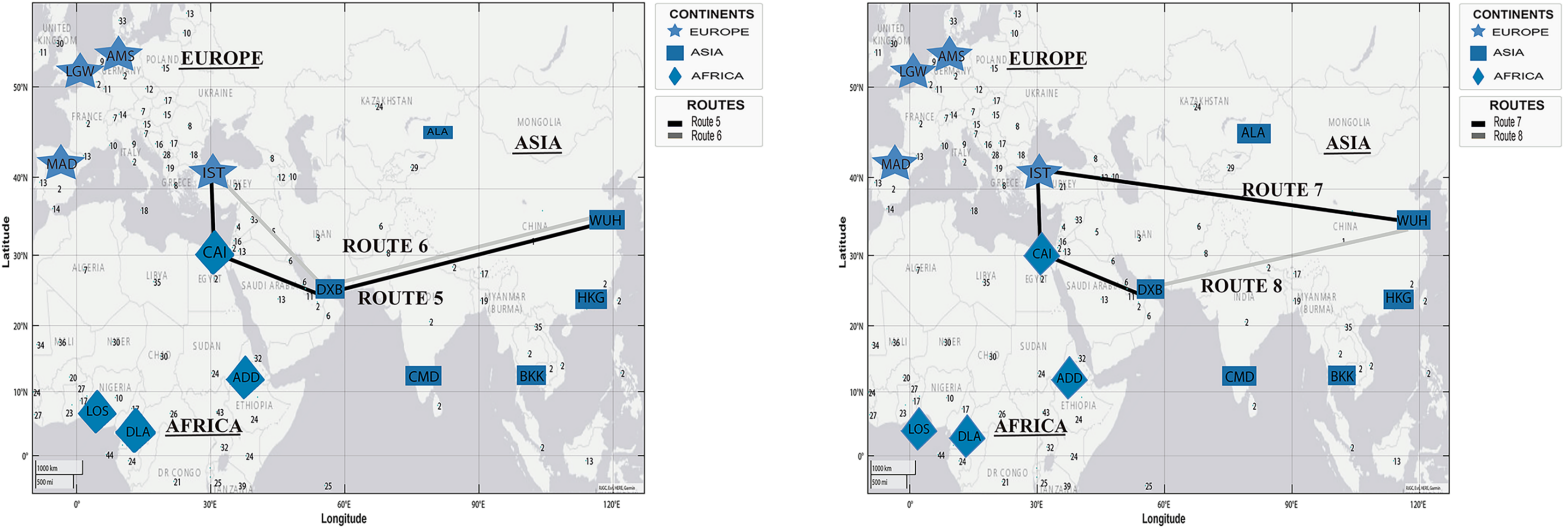
Simulation of COVID-19 spread routes from WHU to Istanbul (Turkey) and Dubai (United Arab Emirates) by applying the DetARPDS algorithm.

Figure 7 presents two simulated routes from WHU to Istanbul (Turkey) and Dubai (United Arab Emirates), based on the temporal information of the spread of the virus in Table 4.

Route 5: WUH–DXB–CAI–IST

- (WUH) WHU-Tianhe International Airport (Group 1)
- (DXB) Dubai International Airport (Group 1)
- (CAI) Cairo International Airport (Group 2)
- (IST) Istanbul Airport (Group 4)

Route 6: WUH–DXB–IST

- (WUH) WHU-Tianhe International Airport (Group 1)
- (DXB) Dubai International Airport (Group 1)
- (IST) Istanbul Airport (Group 4)

Route 7: WUH–IST–CAI–DXB

- (WUH) WHU-Tianhe International Airport (Group 1)
- (IST) Istanbul Airport (Group 4)
- (CAI) Cairo International Airport (Group 2)
- (DXB) Dubai International Airport (Group 1)

Route 8: WUH–DXB

- (WUH) WHU-Tianhe International Airport (Group 1)
- (DXB) Dubai International Airport (Group 1)

**Figure 8:**
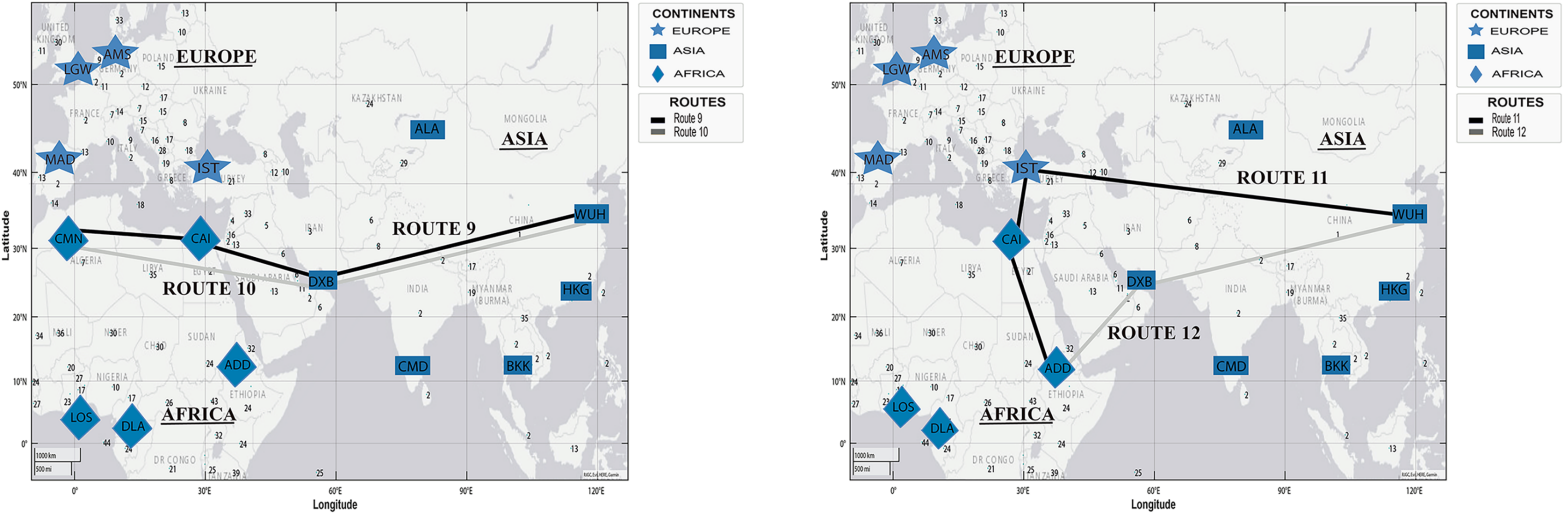
Simulation of COVID-19 spread routes from WHU to Casablanca (Morocco) and Addis Ababa (Ethiopia) by applying the DetARPDS algorithm.

Figure 8 presents two simulated routes from WHU to Casablanca (Morocco) and Addis Ababa (Ethiopia), based on the spread of COVID-19 in Table 4.

Route 9: WUH–DXB–CAI–CMN

- (WUH) WHU-Tianhe International Airport (Group 1)
- (DXB) Dubai International Airport (Group 1)
- (CAI) Cairo International Airport (Group 2)
- (CMN) Mohammed V International Airport (Group 4)

Route 10: WUH–DXB–CMN

- (WUH) WHU-Tianhe International Airport (Group 1)
- (DXB) Dubai International Airport (Group 1)
- (CMN) Mohammed V International Airport (Group 4)

Route 11: WUH–IST–CAI–ADD

- (WUH) WHU-Tianhe International Airport (Group 1)
- (IST) Istanbul Airport (Group 4)
- (CAI) Cairo International Airport (Group 2)
- (ADD) Bole Addis Ababa International Airport (Group 4)

Route 12: WUH–DBX–ADD

- (WUH) WHU-Tianhe International Airport (Group 1)
- (DXB) Dubai International Airport (Group 1)
- (ADD) Bole Addis Ababa International Airport (Group 4)

**Figure 9:**
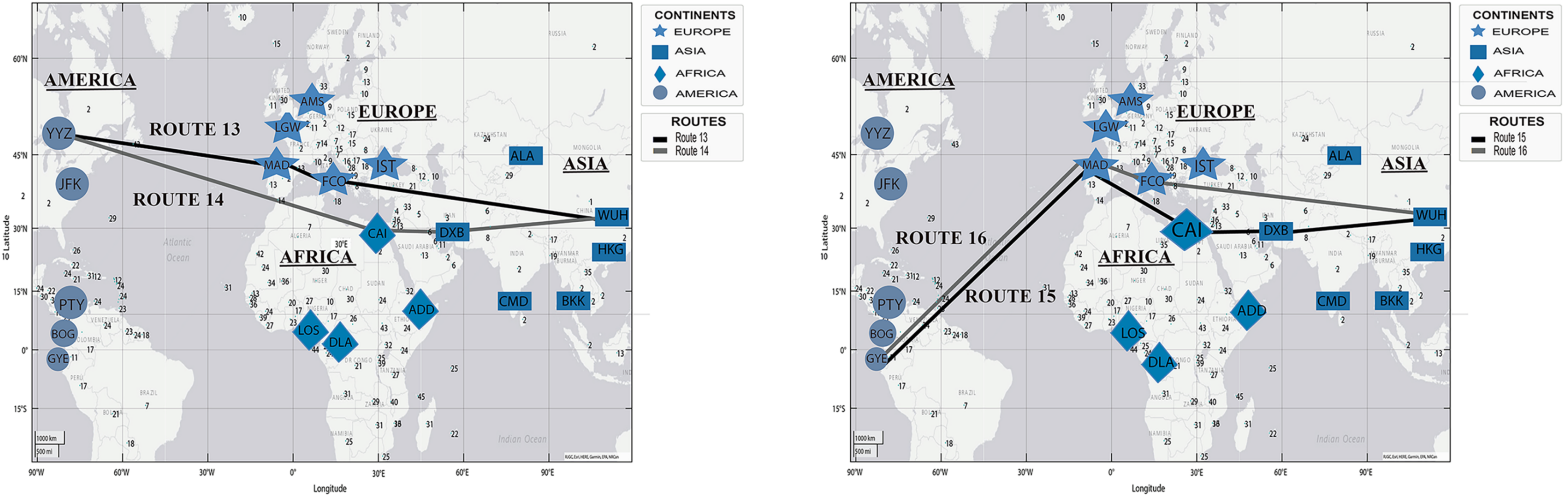
Simulation of COVID-19 spread routes from WHU to Toronto (Canada) and Guayaquil (Ecuador) by applying the DetARPDS algorithm.

Figure 9 presents two simulated routes from WHU to Toronto (Canada) and Guayaquil (Ecuador), based on the spread of COVID-19 in Table 4.

Route 13: WUH–FCO–MAD–YYZ

- (WUH) WHU-Tianhe International Airport (Group 1) p 1)
- (FCO) Rome Fiumicino Airport (Group 1)
- (MAD) Adolfo Suarez Madrid-Barajas Airport (Group 2)
- (YYZ) Toronto Pearson International Airport (Group 1)

Route 14: WUH–DXB–CAI–YYZ

- (WUH) WHU-Tianhe International Airport (Group 1) (Group 1)
- (DXB) Dubai International Airport (Group 1)
- (CAI) Cairo International Airport (Group 2)
- (YYZ) Toronto Pearson International Airport (Group 1)

Route 15: WUH–DXB–MAD–AMS–GYE

- (WUH) WHU-Tianhe International Airport (Group 1) (Group 1)
- (DXB) Dubai International Airport (Group 1)
- (CAI) Cairo International Airport (Group 2)
- (MAD) Adolfo Suarez Madrid-Barajas Airport (Group 2)
- (GYE) Jose Joaquin de Olmedo International Airport (Group 4)

Route 16: WUH–FCO–MAD–GYE

- (WUH) WHU-Tianhe International Airport (Group 1) (Group 1)
- (FCO) Rome Fiumicino Airport (Group 1)
- (MAD) Adolfo Suarez Madrid-Barajas Airport (Group 2)
- (GYE) Jose Joaquin de Olmedo International Airport (Group 4)

**Figure 10:**
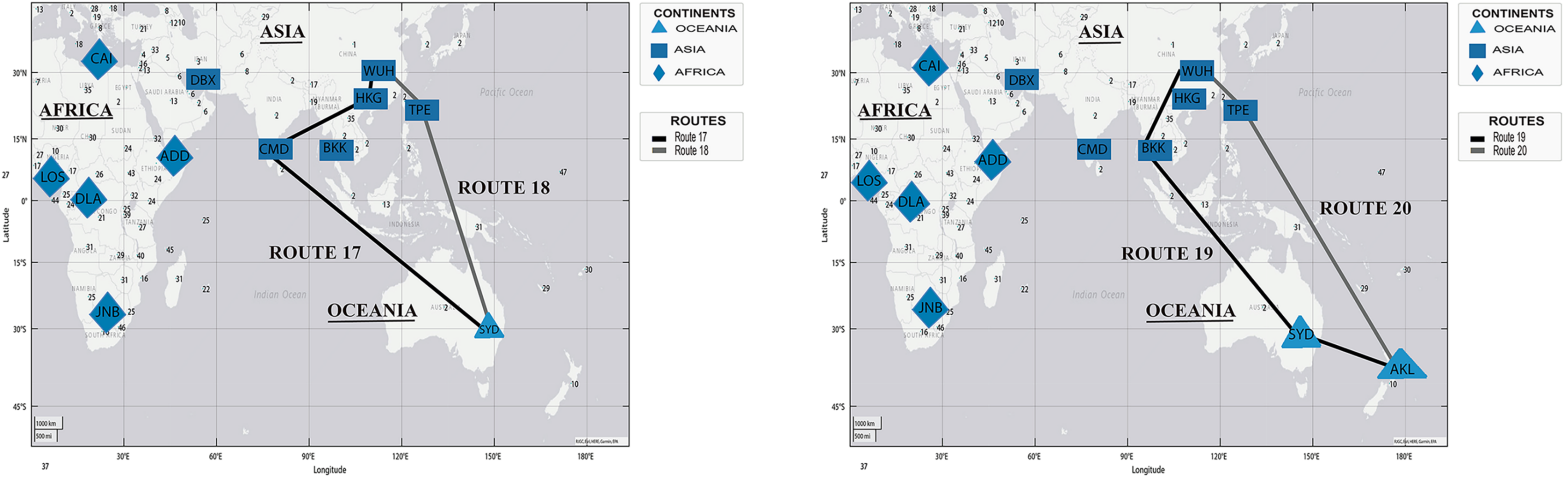
Simulation of COVID-19 spread routes from WHU to Sydney (Australia) and Auckland (New Zealand) by applying the DetARPDS algorithm.

Figure 10 presents two simulated routes from WHU to Sydney (Australia) and Auckland (New Zealand), based on the chronology of COVID-19 spread, in Table 4.

Route 17: WUH–HKG–CMB–SYD

- (WUH) WHU-Tianhe International Airport (Group 1)
- (HKG) Hong Kong International Airport (Group 1)
- (CMB) Bandaranaike International Airport (Group 1)
- (SYD) Sydney Airport (Group 1)

Route 18: WUH–TPE–SYD

- (WUH) WHU-Tianhe International Airport (Group 1)
- (TPE)Taiwan Taoyuan International Airport (Group 1)
- (SYD) Sydney Airport (Group 1)

Route 19: WUH–BKK–SYD–AKL

- (WUH) WHU-Tianhe International Airport (Group 1)
- (BKK) Suvarnabhumi International Airport (Group 1)
- (SYD) Sydney Airport (Group 1)
- (AKL) Auckland International Airport (Group 3)

Route 20: WUH–TPE–AKL

- (WUH) WHU-Tianhe International Airport (Group 1)
- (TPE) Taiwan Taoyuan International Airport (Group 1)
- (AKL) Auckland International Airport (Group 3)

The simulation results showed that the length of connections of the routes ranged from two (two simulated routes) to six terminals (one simulated route), with an average of four connections (11 simulated routes). These simulations present the most likely routes (and closest to reality) for the spread of COVID-19 since the chronology of the spread is evidenced (Table 4), as presented in Table 11.

**Table 11:** Simulations of COVID-19 spread chronology groups.

Route	C1	C2	C3	C4	C5	C6
Route 1	Group 1	Group 1	Group 4	Group 2	Group 3	–
Route 2	Group 1	Group 4	Group 3	–	–	–
Route 3	Group 1	Group 1	Group 4	Group 4	Group 3	Group 1
Route 4	Group 1	Group 4	Group 1	–	–	–
Route 5	Group 1	Group 1	Group 2	Group 4	–	–
Route 6	Group 1	Group 1	Group 4	–	–	–
Route 7	Group 1	Group 4	Group 2	Group 1	–	–
Route 8	Group 1	Group 1	–	–	–	–
Route 9	Group 1	Group 1	Group 2	Group 4	–	–
Route 10	Group 1	Group 1	Group 4	–	–	–
Route 11	Group 1	Group 4	Group 2	Group 4	–	–
Route 12	Group 1	Group 1	Group 4	–	–	–
Route 13	Group 1	Group 1	–	–	–	–
Route 14	Group 1	Group 1	Group 2	Group 1	–	–
Route 15	Group 1	Group 1	Group 2	Group 2	Group 4	–
Route 16	Group 1	Group 1	Group 2	Group 4	–	–
Route 17	Group 1	Group 1	Group 1	Group 1	–	–
Route 18	Group 1	Group 1	Group 1	–	–	–
Route 19	Group 1	Group 1	Group 1	Group 3	–	–
Route 20	Group 1	Group 1	Group 3	–	–	–

The results obtained from the simulations carried out with the DetARPDS algorithm identified probable routes of spread to 10 international air terminals in the five continents around the world, as shown above. These routes contain one or more air terminals with a high audience, where the terminals were detected, as shown in Table 12. To generate each route between the departure airport (start point) and the destination airport (finish point), we applied the information from the international airport network, graph theory, and Dijkstra algorithm. This process obtained many routes with multiple dimensions from one to 10 connections. Algorithm 2 (Table 8) creates the graph of the airport network evaluates all existing possibilities, and identifies the most dangerous ones.

**Table 12:** Information of international air terminals by number of simulations by applying the DetARPDS algorithm.

Airports	R1	R2	R3	R4	R5	R6	R7	R8	R9	R10	Total	%
WUH	2.640	3.391	3.874	3.874	3.874	3.874	3.874	2.373	703	375	28.852	63.46
IST	567	867	3.874	1.680	1.174	1.833	1.428	1.552	558	479	14.012	30.82
DXB	673	642	747	3.874	654	594	668	380	344	282	8.858	19.48
AMS	3.834	105	78	66	111	79	156	1.481	36	33	5.979	13.15
LGW	643	570	1.721	770	811	474	510	340	41	42	5.922	13.02
FRA	83	3.874	91	83	139	189	206	76	41	31	4.813	10.58
YYZ	59	54	68	0	5	52	3.874	40	51	33	4.236	9.31
ADD	75	34	17	27	32	3.874	42	8	33	17	4.159	9.14
CMN	30	37	13	18	3.874	33	19	16	4	3	4.047	8.90
SVO	85	349	660	765	630	652	547	270	14	7	3.979	8.75

The terminal that appeared in the simulations in greater proportion was Wuhan–Tianhe Dubai International Airport (WHU), for being the point of origin of the propagation, followed by Istanbul Airport—(IST) (Fig. 5), which connects with air terminals in Asia and the Middle East, Europe, and Africa. Moreover, Dubai International Airport (DXB), Adolfo Suárez Madrid-Barajas Airport (MAD), and Cairo International Airport (CAI) (Fig. 7) are the main connections from the Middle East to multiple African, European, and Oceanian countries. European terminals, such as AMS, LGW, and FRA, provide several international air routes with direct connections to the Americas, the Middle East, Africa, and Asia. Similarly, on the routes from Asia to Oceania (Fig. 8), it is evident that Taiwan Taoyuan International Airport (TPE) is a common connection. These airports have been identified as almost mandatory connections for routes between continents because they contain a large influx of airline routes and multiple connections to destinations. In the case of spreading a disease, these terminals must be closed or have higher sanitary controls to avoid the spread of the virus to different parts of the world. The remainder of the airports accounted for $\lt \!10$% of the routes simulated by the DetARPDS algorithm, meaning that they had a low concentration of air routes.

Some interesting results presented in Table 11 demonstrate that the simulated routes mainly followed a chronological order of contagion (15 simulated routes 75%) from the origin of the first case of COVID-19 to each of the destination cities. These results indicate that the simulation with the proposed algorithm generated very real routes in the case of spreading a disease in an international air network, which will allow the definition of possible scenarios for decision-making in future health policies.

Table 13 presents the processing times of applying the DetARPDS algorithm to determine the routes of the spread of COVID-19. The results show a maximum time of 780.3960 s, with 6,587 routes generated. However, the average time of 0.11752 s and the minimum of 0.10583 s processing time indicated that the algorithm consumed considerable processing time because it was related to the number of routes generated and nodes contained in the graph. In the simulations carried out, the minimum and maximum times were between 0.10 and 0.19 s for each route generated, where the DetARPDS algorithm searched for the possible routes from the origin and destination terminal in an optimal way. In addition, it was determined that the processing time increased if the route had air terminals with many possible routes, as this increased the number of routes to be generated.

**Table 13:** Processing times of the DetARPDS algorithm when generating COVID-19 spread routes.

Origin $V_i$	Destiny $V_f$	Routes	Min (s)	Mean (s)	Max (s)	Total (s)
WHU	AMS	3.874	0.10329	0.11728	0.19674	461.3999
	FRA	4.625	0.10851	0.11699	0.18799	546.2652
	IST	5.466	0.10773	0.11693	0.18989	643.9816
	DBX	6.587	0.10784	0.11752	0.19386	780.396
	CMN	5.828	0.10259	0.11753	0.19708	691.6327
	ADD	5.787	0.10237	0.11777	0.19679	686.7405
	YYZ	6.138	0.10367	0.11748	0.19111	727.2674
	GYE	3.607	0.10389	0.11868	0.19451	431.7817
	SYD	1.937	0.10947	0.11730	0.18667	229.2843

The results obtained with the DetARPDS algorithm are very similar to the reality of the spread of COVID-19 in the network of airport terminals with 93.46% accuracy (67.534 simulations) in the tests. The 6.53% rest are similar in 47.60% of the routes, where each route had one or more connexions with airports with high infections rate. The literature review found no comparable works where Dijkstra’s algorithm has been applied to COVID-19 in airport networks. The most similar were those presented by Fijar Awalivian, Suyanto & Sa’Adah (2021), Yanzhe & Bingxiang (2020). These studies used genetic algorithms to predict the contagion routes with high accuracy and obtained results similar to those obtained by the DetARPDS algorithm. In the study by Fijar Awalivian, Suyanto & Sa’Adah (2021), the accuracy is variable and depends on the quantity of data which is around 80%. Our proposal uses and analyses a structured network of airports and a small number of instances with optimal results. Another advantage of our algorithm is that it uses the information on the chronology of the spread by verifying the routes simulated and guaranteeing the efficiency of the results. Determining airports with the highest probability of infection is another significant contribution of this study, which was also addressed by Lu et al. (2021) with similar results, where the prediction rate is 90.7% and near 91% in work by Yanzhe & Bingxiang (2020). An Advantage of the DetARPDS is the use and analysis of COVID-19 and airport data, which identifies all possible routes from departure airport to destination airport and compare the density of infections in each airport terminal. In comparison with our proposal, Lu et al. (2021) has the scale and quality of the data as limitations, whereas DetARPDS has an analysis that determines the level of danger of the route.

The results of the techniques were compared based on the processing time, where the DetARPDS algorithm consumed the maximum time (727.36 s) followed by the MALO algorithm Fijar Awalivian, Suyanto & Sa’Adah (2021) and Yanzhe & Bingxiang (2020), with more than 1 h of time processing. Through informal tests, we evaluated other techniques with the same data, such as neural networks, genetic algorithms, and fuzzy logic, where time processing was higher than our proposal, and the prediction result was equal or very similar. Above all, our algorithm used the techniques and methods widely used in the literature to achieve the main objective with optimal results and obtained essential information on preventing the spread of diseases in the international airport network.

## Conclusions and future work

In this research, an extensive literature review was conducted to determine the most widely used algorithms for identifying routes more efficiently. As a result of this scientific review, we could present some algorithms, such as EA, DP, Dijkstra’s algorithm, neural networks, TSP, graph theory, Markov chain, and fuzzy logic. Subsequently, we analyzed the database of the network of international airports and the information on COVID-19 infections in detail. One of the main obstacles in the study was collecting information from each air terminal and its routes since this constitutes considerable information that must be collected manually. The graph theory was eventually selected to graph the airport network, and Dijkstra’s algorithm was employed for the generation of routes to develop DetARPDS.

Thanks to the literature review, we could know the framework of reference for the most important algorithms and techniques used in the research area. The first main contribution of the study was the development of a real graph of international airports, which describes the localization, distance, and connexions between all airports around the world. This graph allows knowing all possible routes that the virus will take. The DetARPDS algorithm generates spread routes in a short time near 300 s, where the distance between the terminals is calculated by applying the Euclidean distance between each airport using latitude and longitude.

The second contribution was a discrete analysis of the generated routes of the spread of COVID-19. It was an essential tool for determining the possible behavior of virus propagation. With the graph of international airports, all possible routes are generated from the origin of the virus (the city where the first case of COVID-19 was reported) to 10 cities on different continents, applying the Dijkstra algorithm. The processing of this phase of the DetARPDS algorithm was optimal, with results between 120 to 220 s.

The third contribution was the creation of a chronology of propagation based on historical data on COVID-19. It allowed identifying the routes with a high probability of infection and the airports with high-risk infection. With high accuracy of 93.46%, the DetARPDS algorithm can also determine all possible routes from departure airport to destination airport terminal. With this information, countries, states, and security institutions will define health safety policies to prevent future diseases of dangerous variants of COVID-19.

For future research, it is proposed to incorporate information from other media (such as social networks and migratory information) to improve the model so that it becomes increasingly closer to reality. Moreover, the application of platforms for the simultaneous execution of tasks would reduce the processing time. Finally, the application of deep neural networks can be used to determine the profiles of travelers infected with the virus.

## Supplemental Information

10.7717/peerj-cs.1228/supp-1 Supplemental Information 1 Matlab code and datasets for the experiment.

## Additional Information and Declarations

### Data Availability

The following information was supplied regarding data availability: The code in Matlab and data (COVID-19 data and flight data of the international airport network) are available in the Supplemental File.
